# Different types of screen time, physical activity, and incident dementia, Parkinson’s disease, depression and multimorbidity status

**DOI:** 10.1186/s12966-023-01531-0

**Published:** 2023-11-03

**Authors:** Hanzhang Wu, Yeqing Gu, Wenxiu Du, Ge Meng, Hongmei Wu, Shunming Zhang, Xuena Wang, Juanjuan Zhang, Yaogang Wang, Tao Huang, Kaijun Niu

**Affiliations:** 1 School of Public Health of Tianjin, University of Traditional Chinese Medicine, 10 Poyanghu Road, West Area, Tuanbo New Town, Jinghai District, Tianjin, 301617 China; 2 Nutritional Epidemiology Institute and School of Public Health, Tianjin Medical University, Tianjin, China; 3 School of Integrative Medicine, Public Health Science and Engineering College, Tianjin University of Traditional Chinese Medicine, Tianjin, China; 4 Institute of Radiation Medicine, Chinese Academy of Medical Sciences & Peking Union Medical College, Baidi Road 238, Tianjin, 300192 China; 5 Department of Toxicology and Sanitary Chemistry, School of Public Health, Tianjin Medical University, Tianjin, China; 6 School of Public Health, Tianjin Medical University, Qixiangtai Road 22, Tianjin, 300070 China; 7 Department of Epidemiology and Biostatistics, School of Public Health, Peking University Health Science Center, Beijing, China; 8 Tianjin Key Laboratory of Environment, Nutrition and Public Health, Tianjin, China; 9 Center for International Collaborative Research On Environment, Nutrition and Public Health, Tianjin, China

**Keywords:** Screen time, Dementia, Parkinson’s disease, Depression, Magnetic resonance imaging

## Abstract

**Background:**

Several previous studies have shown that excessive screen time is associated with an increased prevalence of dementia, Parkinson’s disease (PD), and depression. However, the results have been inconsistent. This study aimed to prospectively investigate the association between different types of screen time and brain structure, as well as the incidence of dementia, Parkinson’s disease, depression, and their multimorbidity status.

**Methods:**

We included 473,184 participants initially free of dementia, PD, and depression from UK Biobank, as well as 39,652 participants who had magnetic resonance imaging (MRI) data. Screen time exposure variables including TV viewing and computer using were self-reported by participants. Cox proportional hazards regression models were used to estimate the association between different types of screen time and the incidence of dementia, Parkinson’s disease, depression, and their multimorbidity status. Multiple linear regression models were used to assess the linear relationship between different types of screen time and MRI biomarkers in a subgroup of participants.

**Results:**

During the follow up, 6,096, 3,061, and 23,700 participants first incident cases of dementia, PD, and depression respectively. For moderate versus the lowest computer uses, the adjusted HRs (95% CIs) were 0.68 (0.64, 0.72) for dementia, 0.86 (0.79, 0.93) for PD, 0.85 (0.83, 0.88) for depression, 0.64 (0.55, 0.74) for dementia and depression multimorbidity, and 0.59 (0.47, 0.74) for PD and depression multimorbidity. The multivariable HRs (95% CIs) for the highest versus the lowest group of TV viewing time were 1.28 (1.17, 1.39) for dementia, 1.16 (1.03, 1.29) for PD, 1.35 (1.29, 1.40) for depression, 1.49 (1.21, 1.84) for dementia and depression multimorbidity, and 1.44 (1.05, 1.97) for PD and depression multimorbidity. Moderate computer using time was negatively associated with white matter hyperintensity volume (*β* = -0.042; 95% CI -0.067, -0.017), and positively associated with hippocampal volume (*β* = 0.059; 95% CI 0.034, 0.084). Participants with the highest TV viewing time were negatively associated with hippocampal volume (*β* = -0.067; 95% CI -0.094, -0.041). In isotemporal substitution analyses, substitution of TV viewing or computer using by equal time of different types of PA was associated with a lower risk of all three diseases, with strenuous sports showing the strongest benefit.

**Conclusion:**

We found that moderate computer use was associated with a reduced risk of dementia, PD, depression and their multimorbidity status, while increased TV watching was associated with a higher risk of these disease. Notably, different screen time may affect the risk of developing diseases by influencing brain structures. Replacing different types of screen time with daily-life PA or structured exercise is associated with lower dementia, PD, and depression risk.

**Supplementary Information:**

The online version contains supplementary material available at 10.1186/s12966-023-01531-0.

## Introduction

Neurodegenerative disorders are incurable diseases that cause progressive degeneration or death of nerve cells and affect approximately one billion people worldwide [1]. Dementia and Parkinson’s disease (PD) are the two most prevalent neurodegenerative diseases, and the global incidence of these diseases are increasing rapidly due to the aging of the population. Dementia is a syndrome in which there is a deterioration in cognitive function that interferes with independent daily functions [2]. Currently, more than 55 million people worldwide live with dementia, and there are nearly 10 million new cases every year [3]. Motor symptoms, such as bradykinesia, muscular rigidity, and a rest tremor of 4–6 Hz, are commonly observed in individuals with PD [4]. In 2016, an estimated 6.1 million individuals globally were diagnosed with PD, which is 2.4 times higher than the number of diagnoses in 1990 [5]. Depression is a common comorbidity of neurodegeneration, affecting an estimated 350 million people worldwide [6]. Mental health costs have considerably exceeded in recent years, and the global cumulative economic loss is estimated to be $5.36 trillion from 2011 to 2030 [7]. Neurodegenerative disorders and depression are comorbid conditions that share some of the same, largely modifiable, risk and protective factors.

Although associations between screen time and dementia have been examined [8, 9], evidence still lack regarding the development of PD, depression, and multimorbidity status. Previous studies have demonstrated that screen time may have effects on cognition and structural brain aging [10, 11]. Specific patterns of brain atrophy, assessed by structural magnetic resonance imaging (MRI), are valid markers of neurodegeneration [12]. Although MRI is widely used in research, the association between different types of screen time and brain volume is sparse and inconclusive.

The health benefits associated with physical activity (PA) have been well-established [13–15]. The latest guidelines on PA and sedentary behavior from the World Health Organization recommend replacing sedentary time with PAs of any intensity, including light-intensity activities, to provide health benefits [16]. Previous studies have revealed that replacing sedentary time with equivalent amounts of PA might be associated with a reduced risk of type 2 diabetes, frailty, depressive symptoms, and mortality [17–20]. However, investigators have typically examined various types of screen time and structured exercises separately, without considering the displacement of time-dependent behaviors.

This study aimed, therefore, to determine the association between different types of screen time and the risk of dementia, PD, depression, and multimorbidity status. Furthermore, using the large-scale brain imaging data from the UK Biobank, we aimed to investigate the association between different types of screen time and brain structure. Finally, we used the same cohort to investigate the association between different types of screen time and PA with the risk of developing dementia, PD, and depression. We assessed different time displacements using the isotemporal substitution model (ISM). We hypothesized that different types of screen time exhibit distinct associations with the risk of these diseases and brain structure. Additionally, we predicted that replacing screen time with PA would reduce the risk of these diseases.

## Methods

### Study population

The UK Biobank is a prospective population-based cohort study that recruited half a million men and women (with a 5.5% response rate) aged 39–72 years from the general population between 2006 and 2010 [21]. Detailed information on the study population of the UK Biobank Study is presented in the Supplementary Methods.

### Assessment of dementia, PD, depression and multimorbidity status

Prevalent and incident cases of dementia and depression within the UK Biobank were identified using inpatient records. These records contained data on admissions and diagnoses obtained from the Hospital Episode Statistics. Diagnoses were recorded using the International Classification of Diseases (version 10; code ICD-10) coding system. We defined outcomes according to the ICD-10: dementia (F00, F01, F02, F05.1, F10.6, G30, G31.0, G31.1, G31.8, A81.0, and I67.3), PD (G20), and depression (F32, F33). Multimorbidity is defined as the presence of two or more chronic diseases occurring simultaneously in an individual. The date of updating the linkages to hospital inpatient admission and death registries was September 20, 2021 in England, July 31, 2021 in Scotland, and February 28, 2018 in Wales for this study.

### Assessment of screen time

In the UK Biobank, relevant screen time exposure variables were assessed through self-reported time spent watching TV and using the computer outside of work. TV viewing time was assessed by asking the following question: “In a typical DAY, how many hours do you spend watching TV? (Put 0 if you do not spend any time doing it)” Daily recreational computer use time was assessed by asking the following question: “In a typical DAY, how many hours do you spend using the computer? (Do not include using a computer at work; put 0 if you do not spend any time doing it)” Durations of less than 0 h or more than 24 h were rejected. If the participants watch TV for more than 8 h or use a computer for more than 6 h, they are asked to confirm. If the respondent replied “Less than an hour a day”, this was recorded as 0.5 h/day.

### Assessment of physical activity

Total PA was assessed using a modified version of the International Physical Activity Questionnaire (IPAQ). Participants were asked to report the frequency and duration of walking, moderate, and vigorous activities on a typical day or week over the past four weeks. This is a validated questionnaire that previous studies have demonstrated reliability and validity [22, 23]. Details on the definition of PA were provided in the Supplementary Methods.

### MRI data acquisition and processing

MRI data were acquired using a Siemens Skyra 3 T scanner (Siemens Healthcare, Erlangen, Germany) with a standard 32-channel head coil. Details of the freely available protocol are available at http://www.fmrib.ox.ac.uk/ukbiobank/protocol/V4_23092014.pdf. The T1 and T2 weighted scans were analyzed using the Functional Magnetic Resonance Imaging of the Brain Software Library. Total brain volume was calculated by adding the volumes of gray matter and white matter. The volumes of the total brain, grey matter, white matter, and white matter hyperintensity (WMH) were normalized for head size [24].

### Assessment of covariates

To mitigate the influence of confounding variables that may distort the associations with incident diseases, we incorporated socio-demographic information, lifestyle factors, and anthropometric measurements as covariates in the analysis. Details on the definition of covariates were provided in the Supplementary Methods.

### Statistical analysis

Person-years of follow-up for each participant in the UK Biobank were calculated from the date of first completing the Oxford WebQ dietary assessment until the date of incident dementia, PD or depression, death, loss to follow-up, or the end of the study. For the follow-up, the status of multimorbidity status was calculated until the date of the incident second disease. The normality of the continuous variables was examined using a Q-Q plot. Baseline characteristics of the study participants were presented as medians (interquartile range) or means (standard deviation) for continuous variables and as percentages for categorical variables, categorized by TV viewing time and computer usage time. We further defined TV viewing time and computer using time into the following categories: 0 to 1, 2, 3, and ≥ 4 h per day for TV viewing, and 0, 0.5 to 1, 2 to 3, and ≥ 4 h per day for computer using. The Cox proportional hazards regression model was used to assess the hazard ratios (HRs) and 95% confidence intervals (CIs) for the incidence of dementia, PD, depression or multimorbidity status across baseline categories of TV viewing time and computer using time. The proportional hazards assumption was tested using Schoenfeld residuals, and no violation of this assumption was found in our analyses. Restricted cubic spline models were used to semi-parametrically investigate the dose–response relationships between TV viewing time and computer using time and the incidence of dementia, PD, depression, and multimorbidity status. More detailed information can be found in the Supplementary Methods.

Multivariable linear regression models were used to examine the association between computer using time or TV viewing time and WMH, hippocampal volume, total brain volume, white matter volume, and gray matter volume. (Supplementary Methods).

In order to further investigate whether engaging in discretionary PA can mitigate the risk of dementia, PD, and depression caused by different types of screen time, we conducted an ISM to evaluate the impact of replacing one type of PA with one type of screen time for an equivalent duration (in this case, 30 min/day) [25] (Supplementary Methods).

The interaction between different categories of screen time and PA was assessed using the likelihood ratio test. This was done by including a multiplicative interaction term in the fully adjusted models. Moreover, to investigate the joint associations of categories of different types of screen time and PA (low, medium, and high) on the development of dementia, PD, and depression, participants were divided into 3 × 4 groups. The reference group consisted of individuals with high levels of PA and the lowest categories of screen time.

In sensitivity analyses, we conducted stratified analyses because sedentary behavior varies by gender and age. Moreover, we excluded individuals who were diagnosed with dementia, PD, or depression within the first 3 years of follow-up to consider the potential influence of reverse causation.

Hazard ratios, along with their corresponding 95% CI were calculated. A two-sided *p* < 0.05 was considered statistically significant. The statistical analyses were performed using SAS version 9.3 (SAS Institute Inc., Gary, NC, USA) and R software version 3.6.1.

## Results

**Fig. 1:**
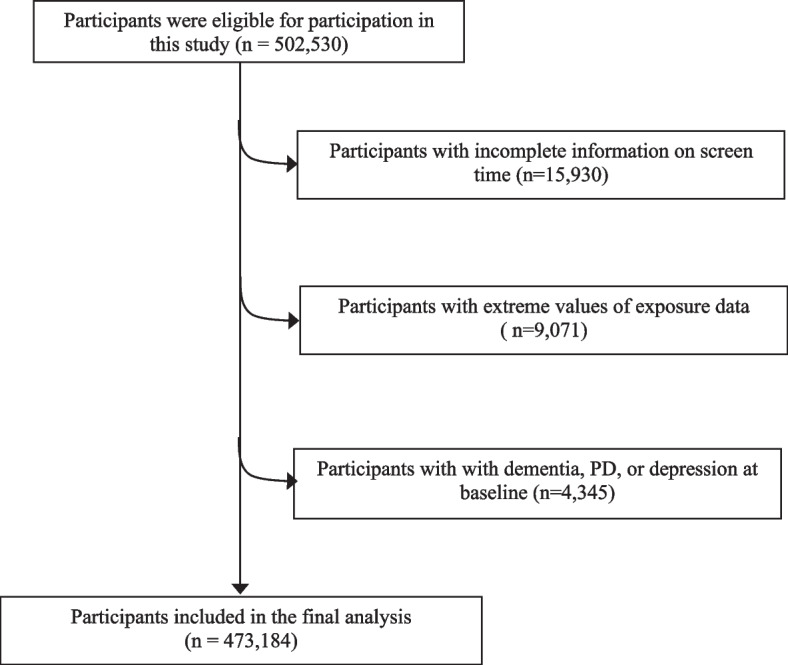
Selection of study participants

In the UK Biobank, we excluded participants with incomplete information on screen time (*n* = 15,930), extreme values of exposure data (abnormal values beyond the mean ± 4 standard deviations: *n* = 9,071), and those with dementia, PD, or depression at baseline (*n* = 4,345). A total of 473,184 participants were finally included in our study (Fig. 1).

In the present study, 6,091 (1.29%) participants developed dementia, 3,054 (0.65%) participants developed PD, 23,632 (4.99%) participants developed depression, 1,214 (0.26%) participants developed both dementia and depression multimorbidity, and 486 (0.10%) participants developed both PD and depression multimorbidity. The baseline characteristics of the study participants from the UK Biobank are shown in Table 1. Participants with the highest TV viewing time were older, more likely to be male, had a higher body mass index (BMI), lower PA and were more likely to be previous or current smokers. Participants with the highest computer using time were younger, more likely to be male, had a higher BMI, lower PA and were previous or current smokers. Table S1 shows the baseline characteristics of participants with MRI data (*n* = 39,652).

**Table 1:** Baseline characteristics of participants by TV viewing time and computer using time in the UK Biobank Study^a^

Characteristics	Computer using time, hours/day				TV viewing time, hours/day			
	0	0.5 to 1	2 to 3	≥ 4	0 to 1	2	3	≥ 4
No. of participants	129,605	238,711	80,675	24,193	97,510	127,448	112,080	136,146
Age (years)	60.0 (12.0)	57.0 (13.0)	58.0 (13.0)	55.0 (14.0)	56.0 (13.0)	56.0 (13.0)	58.0 (12.0)	61.0 (11.0)
Sex (male, %)	38.7	43.4	57.4	60.0	44.3	45.5	45.6	45.6
Race (White, %)	94.3	95.9	92.9	90.3	93.4	94.6	95.3	95.2
BMI (kg/m^2^)	26.9 (5.96)	26.3 (5.46)	27.4 (5.87)	27.6 (6.09)	26.3 (5.40)	26.3 (5.40)	27.0 (5.61)	27.9 (6.07)
Townson depretive index	-1.71 (4.70)	-2.48 (3.57)	-2.06 (4.30)	-1.70 (4.76)	-2.39 (3.75)	-2.39 (3.75)	-2.35 (3.79)	-1.89 (4.53)
PA (MET × hour/week)	1200.0 (3186.0)	1386.0 (2586.0)	1226.5 (2499.0)	1459.0 (2571.0)	1390.0 (2677.5)	1392.0 (2677.5)	1293.0 (2772.0)	1039.5 (2754.0)
Daily-life activities (min/day)	15.0 (39.6)	20.4 (41.8)	18.2 (41.8)	21.4 (42.3)	20.4 (42.9)	0.68 (1.43)	19.3 (40.7)	12.9 (39.6)
Walking for pleasure (min/day)	6.43 (25.7)	6.43 (25.5)	6.43 (25.7)	8.57 (25.0)	6.43 (25.5)	0.23 (0.85)	6.43 (25.7)	6.43 (25.7)
Light DIY (min/day)	0.00 (6.43)	2.14 (12.9)	1.07 (12.9)	3.00 (12.9)	1.61 (12.9)	0.05 (0.43)	0.54 (9.64)	0.00 (6.43)
Heavy DIY (min/day)	0.00 (2.14)	0.00 (6.43)	0.00 (6.43)	0.00 (6.43)	0.00 (6.43)	0.00 (0.21)	0.00 (4.29)	0.00 (3.00)
Structured exercise (min/day)	0.00 (11.6)	3.21 (25.7)	0.75 (17.1)	6.43 (25.7)	3.21 (25.7)	0.11 (0.86)	0.54 (17.1)	0.00 (9.64)
Strenuous sports (min/day)	0.00 (0.00)	0.00 (0.00)	0.00 (0.00)	0.00 (0.00)	0.00 (0.00)	0.00 (0.00)	0.00 (0.00)	0.00 (0.00)
Other exercises (min/day)	0.00 (8.57)	3.20 (21.4)	0.00 (12.9)	4.29 (25.7)	2.14 (21.4)	0.07 (0.71)	0.00 (12.9)	0.00 (8.57)
Smoking status (%)								
Current smoker	13.6	8.01	10.1	12.9	8.19	8.80	9.92	13.0
Ex-smoker	32.6	34.7	38.4	36.9	31.1	33.2	35.8	38.4
Non-smoker	53.8	57.3	51.5	50.2	60.7	58.1	54.3	48.7
Drinking status (%)								
Current drinker	88.9	94.4	92.5	91.5	92.0	93.2	93.3	91.2
Ex-drinker	4.55	2.58	3.75	4.13	3.31	2.91	2.94	4.29
Non-drinker	6.54	3.07	3.79	4.37	4.74	3.86	3.80	4.49
Education level (college or higher, %)	15.3	38.4	41.3	44.9	54.3	39.0	27.6	16.3
Living alone (%)	23.3	15.2	18.7	19.6	18.0	16.3	17.3	21.0
Visiting friends (≥ once a week, %)	50.5	78.9	75.2	71.4	57.7	78.0	79.3	79.8
Healthy diet score (%)								
0–1	6.12	5.11	6.58	7.37	4.27	4.99	5.62	7.64
2–3	43.3	42.2	44.7	44.9	38.5	41.5	43.4	47.5
4–5	50.6	52.7	48.8	47.7	57.2	53.5	51.0	44.9
Individual history of disease (%)								
Hypertension	29.7	22.6	27.8	27.6	18.1	22.4	27.0	33.1
Diabetes	4.69	2.88	4.86	5.49	2.35	2.89	3.78	5.85
Family history of disease (%)								
Dementia	4.09	4.49	4.53	4.34	4.47	4.34	4.38	4.38
Parkinson’s disease	4.16	4.19	4.25	4.00	4.30	4.18	4.14	4.13
Depression	12.4	13.4	13.4	14.2	14.6	13.2	12.7	12.5

*BMI* body mass index, *MET* metabolic equivalent, *MRI* magnetic resonance imaging, *PA* physical activity

^a^Continuous variables are expressed as medians (interquartile range), means [standard deviation], and categorical variables are expressed as percentages

The adjusted association between different types of screen time and risk of dementia, PD, depression, and multimorbidity status is indicated in Table 2. After adjusting for multiple confounders, the multivariable HRs (95% CIs) of dementia across categories of computer using time levels were 1.00 (reference) for 0 h/day, 0.68 (0.64, 0.72) for 0.5–1 h/day, 0.76 (0.70, 0.82) for 2–3 h/day, and 0.77 (0.68, 0.88) for ≥ 4 h/day. In the fully adjusted model, the HR (95% CI) of dementia was 1.28 (1.17, 1.39) for individuals who watched TV for ≥ 4 h/day compared to those who watched TV for 0–1 h/day. In comparisons with participants in the lowest category of computer using time, the HRs (95% CIs) of PD were 0.86 (0.79, 0.93) for 0.5–1 h/day, 0.91 (0.82, 1.01) for 2–3 h/day, and 1.10 (0.94, 1.30) for ≥ 4 h/day, respectively. Participants with the highest TV viewing time had a significantly higher risk (HR = 1.16, 95% CI: 1.03–1.29) for PD compared to those with the lowest TV viewing time (0–1 h/day). For depression, the multivariable HRs (95% CIs) comparing the lowest category of computer using time were 0.85 (0.83, 0.88) for 0.5–1 h/day, 1.00 (0.97, 1.04) for 2–3 h/day, and 1.07 (1.01, 1.13) for ≥ 4 h/day. Participants with the highest TV viewing time had a significantly higher risk (HR = 1.35, 95% CI: 1.29–1.40) for depression compared to those with the lowest TV viewing time (0–1 h/day). In the sensitivity analysis, Table S2 presents results stratified by age and sex subgroups. The associations between different types of screen time and the risk of diseases were generally similar among all subgroups. However, in the subgroup of female and age < 60, the association between the highest TV viewing time and the risk of PD were not statistically significant. The null association between TV viewing time and the risk of PD and depression multimorbidity in all subgroups is likely due to the small number of participants who developed this multimorbidity disease. A similar association between different types of screen time and the risk of dementia, PD, depression, and multimorbidity status was observed after excluding of participants who were diagnosed with these diseases within the first 3 years of follow-up (Table S3). In the multi-adjusted models, a restricted cubic spline model revealed a significant non-linear U-shaped association between computer using time and the risk of dementia, DP, depression, and multimorbidity status (all p-values for non-linearity < 0.001). TV viewing time showed positive associations with dementia, DP, depression, and multimorbidity status in the restricted cubic spline model. Additionally, it exhibited a significant non-linear association with dementia (p for non-linearity = 0.0027), depression (p for non-linearity < 0.0001), and the pooled result (p for non-linearity < 0.0001) (Figures S1). Our results showed that compared to participants who do not use computers, the risk of developing certain conditions generally appeared to decrease with small amounts of computer use. However, the risk then increased again with longer hours of computer use. In contrast, for TV viewing, the risk seemed to increase exponentially with greater exposure.

**Table 2:** Association between different types of screentime and risk of dementia, Parkinson’s disease, depression and multimorbidity status in the UK Biobank Study^a^

	Computer use outside of work (hours/day)				TV viewing time (hours/day)			
	0	0.5 to 1	2 to 3	≥ 4	0 to 1	2	3	≥ 4
**Dementia**								
No. of dementia	2,745	2,097	995	259	788	1,213	1,424	2,671
Person years	1,535,930	2,859,977	951,815	285,271	1,172,953	1,527,799	1,334,984	1,597,257
Incidence per 1000 person years	1.79	0.73	1.05	0.91	0.67	0.79	1.07	1.67
Model 1	1.00 (reference)	0.56 (0.53, 0.60) ^b^	0.65 (0.61, 0.70)	0.70 (0.62, 0.80)	1.00 (reference)	1.04 (0.95, 1.14)	1.16 (1.07, 1.27)	1.51 (1.39, 1.63)
Model 2	1.00 (reference)	0.68 (0.64, 0.72)	0.76 (0.70, 0.82)	0.77 (0.68, 0.88)	1.00 (reference)	1.03 (0.94, 1.13)	1.10 (1.01, 1.21)	1.28 (1.17, 1.39)
**Parkinson’s disease**								
No. of Parkinson’s disease	1,043	1,265	574	179	487	692	725	1,157
Person years	1,538,432	2,860,089	951,822	285,167	1,172,991	1,527,860	1,335,623	1,599,036
Incidence per 1000 person years	0.68	0.44	0.60	0.63	0.42	0.45	0.54	0.72
Model 1	1.00 (reference)	0.84 (0.78, 0.92)	0.92 (0.83, 1.02)	1.14 (0.97, 1.34)	1.00 (reference)	0.98 (0.87, 1.1)	0.99 (0.89, 1.12)	1.11 (1.00, 1.24)
Model 2	1.00 (reference)	0.86 (0.79, 0.93)	0.91 (0.82, 1.01)	1.10 (0.94, 1.30)	1.00 (reference)	1.01 (0.9, 1.13)	1.04 (0.92, 1.17)	1.16 (1.03, 1.29)
**Depression**								
No. of depression	8,136	10,010	4,173	1,381	3,735	5,524	5,433	9,008
Person years	1,503,163	2,817,435	933,926	278,812	1,156,959	1,504,114	1,312,244	1,560,019
Incidence per 1000 person years	5.41	3.55	4.47	4.95	3.23	3.67	4.14	5.77
Model 1	1.00 (reference)	0.69 (0.68, 0.71)	0.88 (0.84, 0.91)	0.98 (0.93, 1.04)	1.00 (reference)	1.09 (1.05, 1.14)	1.20 (1.15, 1.25)	1.62 (1.56, 1.68)
Model 2	1.00 (reference)	0.85 (0.83, 0.88)	1.00 (0.97, 1.04)	1.07 (1.01, 1.13)	1.00 (reference)	1.08 (1.04, 1.13)	1.13 (1.08, 1.18)	1.35 (1.29, 1.40)
**Dementia and Depression Multimorbidity**								
No. of disease	575	380	209	50	141	232	272	569
Person years	1,540,864	2,863,569	953,586	285,734	1,174,372	1,529,909	1,337,485	1,601,987
Incidence per 1000 person years	0.37	0.13	0.22	0.17	0.12	0.15	0.20	0.36
Model 1	1.00 (reference)	0.49 (0.42, 0.56)	0.67 (0.57, 0.80)	0.72 (0.54, 0.98)	1.00 (reference)	1.15 (0.91, 1.44)	1.28 (1.02, 1.59)	1.85 (1.50, 2.26)
Model 2	1.00 (reference)	0.64 (0.55, 0.74)	0.82 (0.68, 0.97)	0.81 (0.60, 1.10)	1.00 (reference)	1.15 (0.92, 1.45)	1.21 (0.96, 1.51)	1.49 (1.21, 1.84)
**Parkinson’s disease and Depression Multimorbidity**								
No. of disease	218	161	81	26	62	95	124	205
Person years	1,541,417	2,863,890	953,736	285,752	1,174,546	1,530,074	1,337,682	1,602,493
Incidence per 1000 person years	0.14	0.06	0.08	0.09	0.05	0.06	0.09	0.13
Model 1	1.00 (reference)	0.53 (0.42, 0.66)	0.68 (0.52, 0.89)	0.96 (0.64, 1.44)	1.00 (reference)	0.97 (0.69, 1.36)	1.27 (0.92, 1.74)	1.48 (1.09, 2.00)
Model 2	1.00 (reference)	0.59 (0.47, 0.74)	0.73 (0.55, 0.96)	0.98 (0.65, 1.49)	1.00 (reference)	1.01 (0.72, 1.42)	1.32 (0.95, 1.82)	1.44 (1.05, 1.97)

Model 1 was adjusted for age, sex, and body mass index

Model 2 was additionally adjusted for race, smoking status, alcohol drinking status, education level, visiting friends, living alone, physical activity, Townson depretive index, healthy dietary score, family history of disease (including dementia, PD, and depression), hypertension, diabetes, and computer using time in TV analysis or TV viewing time in computer analysis

^a^Obtained by using multivariable Cox regression model

^b^Hazard ratios (95% confidence interval) (all such values)

**Fig. 2:**
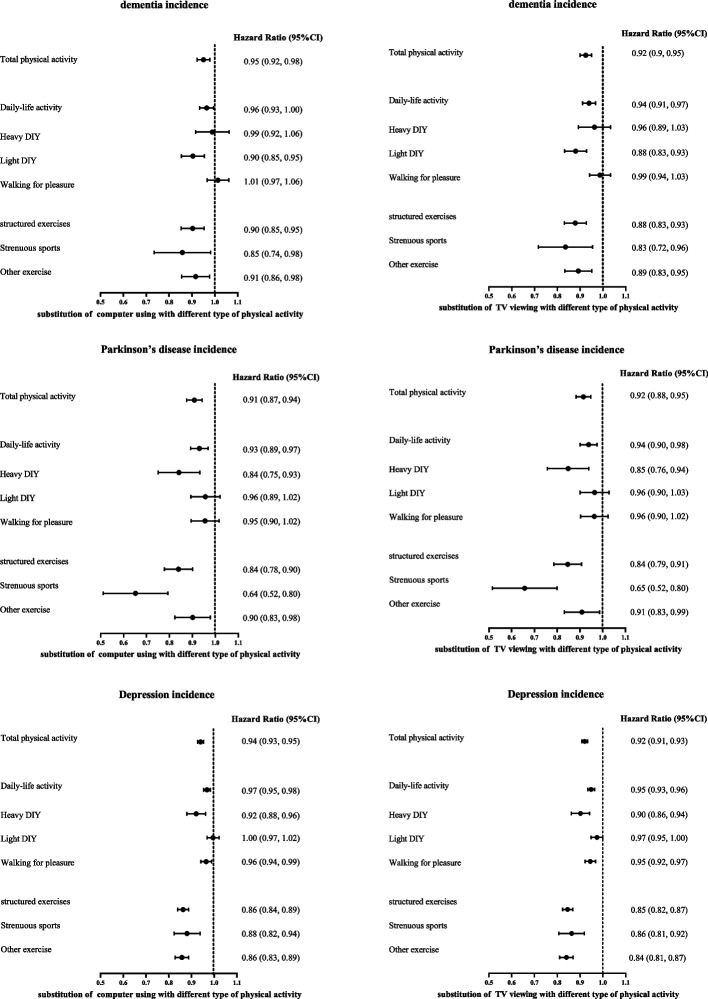
HRs for dementia, Parkinson’s disease and depression according to isotemporal substitution of 30 min/day computer using or TV viewing with equivalent durations of each different type of physical activity. Models are fully adjusted for total discretionary time, time of each type of physical activity and other covariates (Table 2 gives full list of covariates)

We also conducted isotemporal substitution analyses to investigate whether engaging in discretionary PA can mitigate the risk of dementia, PD, and depression induced by excessive computer using time or TV viewing time. Because of the significant non-linear U-shaped association showed protective effect of small amounts of computer using, we excluded people who have computer using time for less than one hour. In the isotemporal substitution analyses, replacing 30 min/day computer using time or TV viewing time with an equal amount of time of different types of activities was associated with significantly lower risks of dementia, PD, and depression (Fig. 2).

Replacing 30 min/day computer using time with an equal amount of time of daily-life activity and structured exercise was associated with 4% (HR 0.96 [95% CI 0.93, 1.00]) and 10% (0.74 [0.85, 0.95]) reductions of dementia risk, 7% (HR 0.93 [95% CI 0.89, 0.97]) and 16% (0.84 [0.78, 0.90]) reductions of PD risk, 3% (HR 0.97 [95% CI 0.95, 0.98]) and 14% (0.86 [0.84, 0.89]) reductions of PD risk, respectively. For replacing 30 min/day TV viewing time, the reduction was 6% (HR 0.94 [95% CI 0.91, 0.97]) and 12% (0.88 [0.83, 0.93]) for dementia risk, 6% (HR 0.94 [95% CI 0.90, 0.98]) and 16% (0.84 [0.79, 0.91]) for PD risk, 5% (HR 0.95 [95% CI 0.93, 0.96]) and 15% (0.85 [0.82, 0.87]) for depression risk, respectively. In assessment of the specific types of PA, the greatest risk reduction was found in modeling 30 min/day reallocations from computer using time or TV viewing time into strenuous sports in all the ISM.

**Fig. 3:**
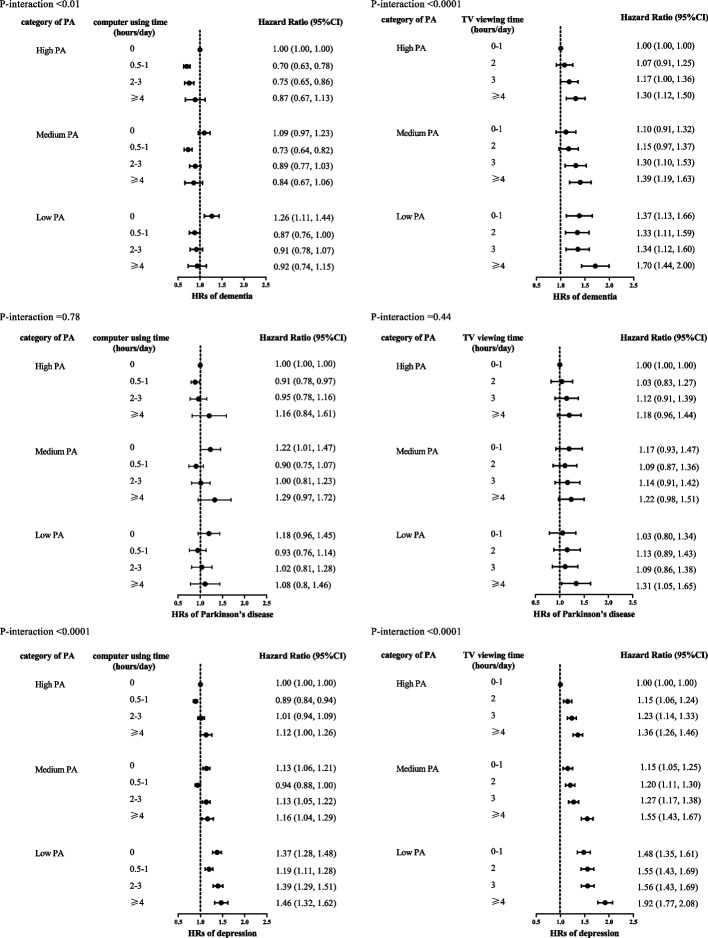
Joint association of PA and computer using or TV viewing with risk of incident dementia, Parkinson’s disease, and depression. Models are fully adjusted (Table 2 gives full list of covariates). Reference categories are the lowest risk for each group (i.e., high PA and low TV watching or high PA and low computer using)

We also found a significant interaction between computer using time or TV viewing time and levels of PA on the risk of incident dementia or depression (all p for interaction < 0.01). Furthermore, we examined joint associations of different types of screen time and levels of PA (Fig. 3). In analyses of these joint associations, with high PA level and low computer using time or TV viewing time as the reference group, there is a clear pattern in which moderate computer using time was associated with decreased disease risk and the highest TV viewing time was significantly associated with increased disease risk across all levels of PA. Moreover, there was substantially increased risk of dementia and depression among those with both low PA and highest TV viewing time.

Table 3 showed the relationship between computer using time or TV viewing time and brain MRI indices. Compared with the lowest group, moderate computer using time was negatively associated with WMH (*β* = -0.042; 95% CI -0.067, -0.017), and positively associated with hippocampal volume (*β* = 0.059; 95% CI 0.034, 0.084). Compared with the lowest group, participants in the highest computer using time were negatively associated with total brain volume (*β* = -0.076; 95% CI -0.117, -0.035), and total white matter volume (*β* = -0.097; 95% CI -0.145, -0.050). Furthermore, compared with the lowest group, participants in the highest TV viewing time were negatively associated with hippocampal volume (*β* = -0.067; 95% CI -0.094, -0.041), and positively associated with total brain volume (*β* = 0.072; 95% CI 0.041, 0.097) and total gray matter volume (*β* = 0.095; 95% CI 0.073, 0.118). Figures S2 showed the non-linear effects of computer using time or TV viewing time on MRI indices. Our results showed that compared to participants who do not use computers, small amounts of computer using seemed to be negatively associated with WMH and positively associated with hippocampal volume. As computer using time increases, the *β* for WMH approaches zero, while the *β* for hippocampal volume reaches a platform stage. For TV viewing, the *β* for hippocampal volume seems to decrease exponentially with exposure, and the increase of *β* for total brain volume and gray matter volume started to decelerate.

**Table 3:** Association between different types of screentime with brain MRI indices in the UK Biobank Study^a^

MRI indices ^b^	Computer use outside of work, hours/day (0 is reference)			TV viewing time, hours/day (0 to 1 is reference)		
	0.5 to 1	2 to 3	≥ 4	2	3	≥ 4
**White matter hyperintensity volume**						
Model 1	-0.055 (-0.080, -0.030) ^c^	-0.019 (-0.049, 0.011)	-0.015 (-0.058, 0.028)	-0.002 (-0.025, 0.020)	0.009 (-0.016, 0.034)	0.026 (0.000, 0.052)
Model 2	-0.042 (-0.067, -0.017)	-0.015 (-0.045, 0.015)	-0.018 (-0.061, 0.025)	-0.006 (-0.028, 0.017)	-0.004 (-0.029, 0.021)	0.002 (-0.024, 0.029)
**Hippocampal volume**						
Model 1	0.090 (0.065, 0.115)	0.077 (0.047, 0.107)	0.051 (0.008, 0.094)	-0.020 (-0.042, 0.003)	-0.045 (-0.07, -0.021)	-0.108 (-0.134, -0.082)
Model 2	0.059 (0.034, 0.084)	0.055 (0.025, 0.085)	0.034 (-0.009, 0.077)	-0.009 (-0.032, 0.013)	-0.022 (-0.047, 0.003)	-0.067 (-0.094, -0.041)
**Total brain volume**						
Model 1	-0.013 (-0.036, 0.011)	-0.048 (-0.076, -0.020)	-0.107 (-0.148, -0.066)	0.048 (0.026, 0.069)	0.070 (0.047, 0.093)	0.090 (0.065, 0.114)
Model 2	0.002 (-0.022, 0.026)	-0.022 (-0.050, 0.007)	-0.076 (-0.117, -0.035)	0.034 (0.013, 0.056)	0.052 (0.028, 0.075)	0.072 (0.047, 0.097)
**Total white matter volume**						
Model 1	-0.022 (-0.049, 0.006)	-0.052 (-0.085, -0.019)	-0.116 (-0.163, -0.069)	0.022 (-0.002, 0.047)	0.036 (0.009, 0.063)	0.039 (0.011, 0.067)
Model 2	-0.010 (-0.038, 0.017)	-0.036 (-0.069, -0.003)	-0.097 (-0.145, -0.050)	0.010 (-0.015, 0.035)	0.017 (-0.010, 0.044)	0.017 (-0.013, 0.046)
**Total gray matter volume**						
Model 1	-0.001 (-0.023, 0.021)	-0.029 (-0.055, -0.003)	-0.065 (-0.103, -0.028)	0.054 (0.034, 0.073)	0.076 (0.055, 0.098)	0.104 (0.081, 0.126)
Model 2	0.012 (-0.010, 0.033)	-0.003 (-0.029, 0.023)	-0.034 (-0.071, 0.003)	0.044 (0.025, 0.063)	0.065 (0.043, 0.086)	0.095 (0.073, 0.118)

Model 1 was adjusted for age, sex, and body mass index

Model 2 was additionally adjusted for race, smoking status, alcohol drinking status, education level, visiting friends, living alone, physical activity, Townson depretive index, healthy dietary score, family history of disease (including dementia, PD, and depression), hypertension, diabetes, and computer using time in TV analysis or TV viewing time in computer analysis

^a^Obtained by using linear regression model

^b^MRI indices were transformed into Z-scores

^c^Beta coefficients (β) (95% confidence interval) (all such values)

## Discussion

The main novel finding of this study is that TV viewing time was positively associated with an increased risk of dementia, PD, depression and their multimorbidity status, while computer using time showed a U-shaped association with a minimum risk at 0.5–1 h/day. These associations remained significant even after considering various traditional risk factors. Furthermore, moderate computer using was found to have a negative association with WMH and a positive association with hippocampal volume. On the other hand, TV viewing was found to have a negative association with hippocampal volume. Moreover, we also found that replacing 30 min/day TV viewing or computer using with an equal amount of time engaging in different types of PA was associated with a lower risk of dementia, PD, and depression. Strenuous sports showed the strongest benefit.

In the present study, engaging in extended periods of computer using or TV viewing is associated with a higher risk of dementia, PD, and depression. Previous studies have shown that sedentary behavior is associated with lower cognitive performance [26]. Furthermore, the results from the English Longitudinal Study of Ageing revealed that TV viewing time was associated with higher levels of depression among a cohort of community-dwelling older adults [27]. In observational studies, television viewing and computer using often serve as indicators of sedentary behaviors [28, 29]. These activities are considered to have low-energy expenditure and have been associated with increased rates of metabolic syndrome, type 2 diabetes, obesity, and cardiovascular disease [30]. The link between cardiovascular health and and the risk of dementia and PD risk has been well established [31, 32]. In addition, sedentary behavior is also associated with biomarkers of low-grade inflammation [33] and changes in inflammation markers that could initiate or worsen neuroinflammation and contribute to neurodegeneration. This process might contribute to dementia, PD, and depression [34–36]. Furthermore, individuals who spend more time watching TV may be characterized by their preference for solitary, non-social leisure activities. This can lead to social isolation and limit the development of social support networks [37]. According to current research results, social support networks appear to have a protective effect on the occurrence of depression [38]. Participants who engage in excessive computer use may spend a significant amount of time on the Internet, especially on social media platforms. This has prompted some individuals to advocate for the recognition of “Internet addiction” [39, 40]. Previous studies have shown a significantly increased risk of developing clinical depression among participants who report moderate or severe Internet addiction [41, 42].

In the present study, moderate computer using time (i.e., approximately 0.5–1 h/day) is associated with a lower risk of dementia, PD, and depression. Although the biological pathways underlying the observed negative association have not been fully elucidated, there is some evidence that may explain the association. First, compared to TV viewing, computer using may require muscular activity, and users are not consistently still when using a computer. Thus, energy expenditure during TV viewing might be lower than during computer use [43] which may result in a positive association between TV viewing and cardiometabolic risk, but a negative association for moderate computer use [44]. Ultimately, the process might influence brain health [31, 32, 45]. Second, individuals who increas their engagement in cognitively stimulating leisure activities, such as computers usage, have a reduced risk of cognitive decline [27, 46]. Third, more people choose are choosing computer for social interaction [47]. Online communication with friends and family increases one’s perceived social support and reduces feelings of loneliness and social isolation, lowering depression and improving mental health [48].

Interestingly, the associations between different types of screen time and dementia, PD, and depression are not strongly attenuated with high levels of PA. However, isotemporal substitution analyses showed that replacing different types of screen time with different types of daily-life physical activities or structured exercises could significantly reduce dementia, PD, and depression risk. Most of the previous studies separately consider the effects of different activities on the risk of dementia, PD, and depression. However, they failed to reflect the competing nature of different activities within a finite leisure time [8, 49]. Moreover, the limited available studies considered TV viewing time and computer using time as representative of all sedentary behavior without considering the heterogeneity of sedentary behavior types or different types of PA [9, 50]. In the isotemporal substitution analyses, we found that PA intensity seemed important in protecting against dementia, PD, and depression because 30 min/day of strenuous sports was more protective than any other PA when substituted for 30 min/day of TV viewing or computer using. However, our results also indicated that not all activities of daily living can protect against dementia, PD, and depression, even if the same number of different types of screen time are replaced. Nevertheless, structured exercises, whether strenuous sports that make one sweat or breathe hard, or other activities such as swimming, cycling, and keeping fit, were associated with protection against dementia, PD, and depression. Our findings complement the PA recommendations that substituting effect of TV viewing or computer using with PA on the risks of dementia, PD, and depression, rather only focusing on separate effects of sedentary time and PA. Our study highlighted that replacing different types of screen time with any equivalent amounts of structured exercises than daily-life activity was more associated with risk reduction of dementia, PD, and depression.

Our results showed that, moderate computer using was negatively associated with WMH, which is common MRI findings in patients with neurodegenerative diseases [51]. Moreover, moderate computer using was positively associated with hippocampal volume, whereas TV viewing was negatively associated. Previous studies have suggested significant reductions of hippocampal volume in patients with dementia, PD, or depression [52–54]. According to our analyses, higher computer using was associated with lower total brain volume and total white matter volume. Higher computer using was associated with higher total brain volume and total gray matter volume. However, this part of the analysis uses a cross-sectional design. Prospective cohort studies are therefore necessary to evaluate the longitudinal association.

The strengths of the current study include its large sample sizes, prospective study design, long-term follow-up, and detailed lifestyle information. We also adjusted for various potential confounding factors and performed a sensitivity analysis by excluding cases of dementia, PD, depression and multimorbidity status that developed within the first 3 years of follow-up. However, several limitations need to be considered when interpreting our results. First, although we have carefully controlled for the potential confounding factors including demographic, lifestyle, and health characteristics that linked with increased risk of these disease, we cannot exclude the possibility of unmeasured factors and residual confounding. Second, screen behaviors were assessed using self‐report measures, so recall bias cannot be avoided. Self-reported screen time has not been examined for criterion validity, but the estimated effectiveness of the screen time reported in this study were in line with those reported previously in comparable populations [9, 55]. However, it is important to note that only TV viewing and leisure time computer use were assessed, despite the existence of other sedentary behaviors such as laptop, tablet, or smartphone usage. The assessment of TV viewing and computer using on weekdays and weekends was not conducted separately in our study. More objective and device‐based measurements to access the screen behavior on weekdays and weekend days separately are needed in the future. Finally, due to the observational nature of this study, we cannot assume causality for the observed association.

## Conclusion

In summary, computer using was U-shaped associated with risk of dementia, PD, depression and their multimorbidity status, while TV viewing was associated with an increased risk. And different screen time may affect diseases risk through its association with brain structures. Although associations between different types of screen time and diseases are not strongly attenuated with different levels of PA. Replacing different types of screen time with daily-life PA or structured exercise is associated with lower disease risk. Our results support the potential of limiting different types of screen time and shifting to physical activity to mitigate disease risk.

### Supplementary Information

**Additional file 1.**

## Data Availability

UK Biobank data are available in a public, open-access repository. This research has been conducted using the UK Biobank Resource under Application Number 44430. The UK Biobank data are available on the application to the UK Biobank (www.ukbiobank.ac.uk/).

## Abbreviations

BMI: Body mass index
CI: Confidence interval
HR: Hazard ratio
ICD: International Classification of Diseases
IPAQ: International Physical Activity Questionnaire
ISM: Isotemporal substitution model
MET: Metabolic equivalent
MRI: Magnetic resonance imaging
PA: Physical activity
PD: Parkinson’s disease
WMH: White matter hyperintensity
