# Supplementary material for: Different types of screen time, physical activity, and incident dementia, Parkinson’s disease, depression and multimorbidity status
Source: Int J Behav Nutr Phys Act. 2023 Nov 3;20:130. doi: 10.1186/s12966-023-01531-0 (PMC10625186; doi:10.1186/s12966-023-01531-0)
Supplement: Supplementary file 1 — Additional file 1. [file 12966_2023_1531_MOESM1_ESM.docx]

**Supplementary Data**

The Association of different types of screen time with brain structure and incident dementia, Parkinson’s disease, depression and multimorbidity status.

**Pages 2-5: Supplementary Methods**

**Pages 6-8: Table S1**. Association between different types of screentime and risk of dementia, Parkinson’s disease, depression and multimorbidity status in the UK Biobank Cohort Study (excluded dementia, Parkinson’s disease, depression, or multimorbidity status that occurred in the first 3 years)

**Pages 9-10: Table S2**. Association between different types of screentime and risk of dementia, Parkinson’s disease, depression and multimorbidity status in the UK Biobank Study (stratified by age and sex).

**Pages 11-13: Table S3**. Association between different types of screentime and risk of dementia, Parkinson’s disease, depression and multimorbidity status in the UK Biobank Study (excluded dementia, Parkinson’s disease or depression that occurred in the first 3 years)

**Page 14-19: Figure S1.** The associations between different types of screen time and incident dementia, Parkinson’s disease, depression and multimorbidity status using a restricted cubic spline regression model.

**Page 20-25: Figure S2.** The associations between different types of screen time with brain MRI indices using a restricted cubic spline regression model.

**Supplementary Methods**

**Study population**

Participants attended one of the 22 assessment centers across England, Wales, and Scotland. The data includes detailed information on participant demographics, social factors, lifestyle, PA, medical history, hospital records, and mortality data. Anthropometric measurements, as well as blood and urine samples, were taken using standardized procedures [1]. It was approved by the research ethics committee of UK Biobank and the Human Tissue Authority Research Tissue Bank, and obtained electronic signed consent from the participants. All participants agreed to use their anonymous data and samples to conduct health-related studies and to reconnect for further sub-studies.

**Assessment of physical activity**

The PA scores were calculated by multiplying the metabolic equivalents (METs) for each type of PA by the minutes performed per week and then combining these measurements. And participants were classified into three levels of PA - ‘low’, ‘medium’, and ‘high’ following IPAQ guidelines [2]. The criteria for these levels have been set, taking into account that IPAQ asks questions in all domains of daily life.

**Categorical Score - three levels of physical activity are proposed**

**1. Low**

• No activity is reported OR

• Some activity is reported but not enough to meet Categories 2 or 3.

**2. Moderate**

Either of the following 3 criteria

• 3 or more days of vigorous activity of at least 20 minutes per day OR

• 5 or more days of moderate-intensity activity and/or walking of at least 30 minutes

per day OR

• 5 or more days of any combination of walking, moderate-intensity or vigorous-intensity activities achieving a minimum of at least 600 MET-minutes/week.

**3. High**

Any one of the following 2 criteria

• Vigorous-intensity activity on at least 3 days and accumulating at least 1500

MET-minutes/week OR

• 7 or more days of any combination of walking, moderate- or vigorous-intensity

activities accumulating at least 3000 MET-minutes/week

For discretionary PA, the frequency and duration of five activities in the previous four weeks was assessed by the touchscreen questionnaire at baseline. Discretionary PA including walking for pleasure, light DIY (do-it-yourself, i.e., home maintenance and improvement and gardening activities), heavy DIY (e.g., using heavy tools, weeding, lawn mowing, digging, carpentry), strenuous sports (i.e., sports that make you sweat or breathe hard), and other activities (e.g., swimming, cycling, keep fit, bowling). Walking for pleasure, light DIY, and heavy DIY were combined to as daily-life activities, and strenuous sports and other exercises were combined to structured exercise. Total PA was calculated by summing five types of discretionary PA.

**Assessment of covariates**

The baseline questionnaire was used to assess several potential confounding variables, including age, sex, self-reported race (white/non-white), smoking status (current smoker, former smoker, never smoked), alcohol drinking status (current drinker, former drinker and never drank), PA, education (college or university degree/ lower) whether they are living alone, and frequency of visiting friends. We also collect information about comorbidities and the patient's family history of the disease. The Townsend Deprivation Index was derived from the postcode of residence using aggregated data on unemployment, car and homeownership, and household overcrowding [3]. The healthy diet score was calculated using the following factors: consuming at least four tablespoons of vegetables per day (median); consuming at least three pieces of fruit per day (median); consuming fish at least twice a week (median); consuming unprocessed red meat no more than twice a week (median); and consuming processed meat no more than twice a week (median). Each point was awarded for each favorable dietary factor, resulting in a total diet score ranging from 0 to 5.

**Statistical Analysis**

Restricted cubic spline models were used to semi-parametrically investigate the non-linear associations between TV viewing time and computer using time and the occurrence of dementia, PD, depression, and multimorbidity status. The models included 4 knots positioned at the 25th, 50th, 75th, and 95th centiles. The departure from linearity of the final cubic spline model was assessed using the Wald test for non-linearity. The p-value for nonlinearity was calculated by testing the null hypothesis that the coefficient of the second spline is equal to 0 [4, 5]. The spline models are fully adjusted, just like the multivariable-adjusted models in the Cox analysis.

Multivariable linear regression models were used to examine the association between computer using time or TV viewing time and WMH, hippocampal volume, total brain volume, white matter volume, and gray matter volume. Results from regression analyses were presented as regression coefficients (β) and their corresponding 95% confidence intervals (CIs). Given the skewed distribution, WMH were log-transformed in the analysis. All MRI biomarkers were converted into Z-scores. To assess the dose-response association between computer using time or TV viewing time and MRI biomarkers, we employed restricted cubic spline functions with four knots positioned at the 25th, 50th, 75th, and 95th centiles.

The ISM was expressed as a basic proportional hazards model, as follows:

*h* (*t)*= *h_0_* (*t)* exp (β_1_ walking for pleasure + β_2_ light DIY + β_3_ heavy DIY + β4 strenuous exercise + β_5_ other exercises + β_6_ computer using time or TV viewing time + β_7_ total discretionary time + β_8_ covariates).

The total discretionary time = computer using time + TV viewing time + total PA time. Considering the “leisure time physical activity” based public health recommendation of 30 minutes on most days will be achieved by most adults in a population, the present study sets 30 min/day as 1 activity component at a time. By virtue of eliminating the computer using time or TV viewing time, the coefficient (β1) represents the effect of substituting 30 min/day TV viewing time or computer using time with 30 min/day walking for pleasure, the coefficient (β2) represents the effect of substituting with 30 min/day light DIY, and so on [6].

| **Table S1. Baseline characteristics of participants with MRI data by TV viewing time and computer using time in the UK Biobank Study ^a^** | | | | | | | | |
| --- | --- | --- | --- | --- | --- | --- | --- | --- |
| Characteristics | Computer using time, hours/day | | | | TV viewing time, hours/day | | | |
|  | 0 | 0.5 to 1 | 2 to 3 | ≥4 | 0 to 1 | 2 | 3 | ≥4 |
| No. of participants | 5,777 | 23,950 | 7,743 | 2,182 | 10,778 | 12,181 | 8,860 | 7,833 |
| Age (years) | 56.0 (12.0) | 55.0 (12.0) | 57.0 (12.0) | 55.0 (12.0) | 54.0 (12.0) | 54.0 (12.0) | 56.0 (11.0) | 58.0 (10.0) |
| Sex (male, %) | 33.8 | 44.3 | 60.0 | 61.9 | 45.3 | 46.6 | 48.2 | 47.5 |
| Race (White, %) | 96.9 | 97.6 | 95.7 | 93.9 | 96.4 | 97.0 | 97.3 | 97.0 |
| BMI (kg/m^2^) | 25.7 (5.18) | 25.7 (4.97) | 26.7 (5.31) | 26.9 (5.59) | 25.0 (4.70) | 25.7 (4.97) | 26.5 (5.06) | 27.2 (5.50) |
| Townson depretive index | -2.44 (3.54) | -2.71 (3.21) | -2.45 (3.71) | -2.42 (4.08) | -2.5.0 (3.64) | -2.68 (3.26) | -2.69 (3.24) | -2.55 (3.43) |
| PA (MET × hour/week) | 1399.5 (2897.5) | 1447.5 (2424) | 1353.0 (2318.0) | 1130.8 (2019.0) | 1491.0 (2293.0) | 1426.0 (2421.0) | 1381.3 (2480.0) | 1279.5 (2572.5) |
| Daily-life activities (min/day ) | 27.9 (47.4) | 30.0 (46.1) | 30.0 (49.3) | 25.7 (40.7) | 30.0 (46.1) | 30.0 (46.1) | 28.9 (47.1) | 27.5 (48.2) |
| Walking for pleasure (min/day ) | 9.64 (22.5) | 9.64 (22.5) | 9.64 (22.5) | 8.57 (22.5) | 9.64 (22.5) | 9.64 (22.5) | 9.64 (22.5) | 9.64 (22.5) |
| Light DIY (min/day ) | 4.29 (12.9) | 6.43 (20.4) | 6.43 (20.7) | 6.43 (12.9) | 8.57 (20.4) | 6.43 (18.5) | 6.43 (19.3) | 5.00 (12.9) |
| Heavy DIY (min/day ) | 1.07 (8.57) | 3.00 (9.64) | 3.00 (9.64) | 1.07 (8.57) | 3.21 (9.64) | 3.00 (9.64) | 2.14 (8.57) | 1.07 (8.57) |
| Structured exercise (min/day ) | 8.60 (25.7) | 12.9 (33.3) | 8.60 (25.7) | 8.60 (32.1) | 12.9 (36.4) | 12.9 (33.3) | 8.60 (25.7) | 6.40 (25.7) |
| Strenuous sports (min/day ) | 0.00 (0.00) | 0.00 (0.00) | 0.00 (0.00) | 0.00 (0.00) | 0.00 (3.21) | 0.00 (0.00) | 0.00 (0.00) | 0.00 (0.00) |
| Other exercises (min/day ) | 6.43 (25.7) | 8.57 (25.7) | 6.43 (25.7) | 6.43 (25.7) | 8.57 (24.6) | 8.57 (25.7) | 6.43 (25.7) | 4.29 (25.7) |
| Smoking status (%) |  |  |  |  |  |  |  |  |
| Current smoker | 61.9 | 62.6 | 57.4 | 55.9 | 64.6 | 63.1 | 59.1 | 55.5 |
| Ex-smoker | 30.9 | 32.0 | 36.2 | 35.4 | 29.6 | 31.5 | 34.7 | 37.3 |
| Non-smoker | 7.14 | 5.45 | 6.42 | 8.77 | 5.77 | 5.41 | 6.25 | 7.29 |
| Drinking status (%) |  |  |  |  |  |  |  |  |
| Current drinker | 93.6 | 96.3 | 94.8 | 94.4 | 94.8 | 96.1 | 95.9 | 95.3 |
| Ex-drinker | 2.46 | 1.73 | 2.51 | 2.80 | 2.26 | 1.88 | 1.85 | 2.24 |
| Non-drinker | 3.95 | 1.94 | 2.69 | 2.80 | 2.93 | 2.07 | 2.22 | 2.52 |
| Education level (college or higher, %) | 28.9 | 48.8 | 52.6 | 54.2 | 64.3 | 50.0 | 38.7 | 27.7 |
| Living alone (%) | 18.2 | 13.8 | 16.9 | 17.0 | 15.7 | 14.4 | 14.7 | 16.5 |
| Visiting friends (≥ once a week, %) | 79.7 | 78.0 | 74.3 | 94.4 | 75.2 | 76.7 | 78.7 | 78.9 |
| Healthy diet score (%) |  |  |  |  |  |  |  |  |
| 0-1 | 4.58 | 4.65 | 5.87 | 6.67 | 3.62 | 4.68 | 5.61 | 6.66 |
| 2-3 | 40.5 | 41.8 | 44.2 | 43.3 | 37.8 | 41.6 | 43.8 | 47.2 |
| 4-5 | 54.9 | 53.6 | 50.0 | 50.1 | 58.6 | 53.7 | 50.6 | 46.2 |
| Individual history of disease (%) |  |  |  |  |  |  |  |  |
| Hypertension | 18.2 | 17.0 | 21.9 | 22.3 | 13.7 | 16.7 | 21.1 | 24.7 |
| Diabetes | 1.90 | 1.67 | 2.96 | 3.12 | 1.27 | 1.61 | 2.28 | 3.47 |
| Family history of disease (%) |  |  |  |  |  |  |  |  |
| Dementia | 4.61 | 4.64 | 4.42 | 5.13 | 4.42 | 4.42 | 4.59 | 5.26 |
| Parkinson’s disease | 4.44 | 4.08 | 4.06 | 5.02 | 4.33 | 3.93 | 4.36 | 4.16 |
| Depression | 13.2 | 14.2 | 14.6 | 15.5 | 15.1 | 13.9 | 14.1 | 13.6 |
| MRI indices |  |  |  |  |  |  |  |  |
| White matter hyperintensity volume (ml) | 2.96 (4.40) | 2.77 (4.11) | 3.22 (5.08) | 2.91 (4.61) | 2.55 (3.88) | 2.71 (4.05) | 3.07 (4.51) | 3.46 (5.15) |
| Hippocampal volume (ml) | 7.33 [0.78] | 7.47 [0.77] | 7.49 [0.79] | 7.53 [0.77] | 7.51 [0.78] | 7.50 [0.77] | 7.43 [0.78] | 7.33 [0.78] |
| Total brain volume (ml) | 1495.8 [73.5] | 1495.2 [73.0] | 1482.4 [73.8] | 1484.7 [76.7] | 1495.5 [73.8] | 1496.6 [74.0] | 1489.9 [72.9] | 1483.5 [72.9] |
| Total white matter volume (ml) | 700.5 [40.5] | 701.1 [40.6] | 699.3 [40.8] | 699.1 [42.1] | 701.2 [40.5] | 701.8 [40.7] | 700.3 [40.9] | 698.1 [40.8] |
| Total grey matter volume (ml) | 795.3 (66.9) | 794.1 (65.1) | 782.9 (64.9) | 783.9 (66.4) | 794.2 (64.9) | 795.1 (66.5) | 789.5 (64.9) | 785.1 (65.7) |
| BMI, body mass index; MET, metabolic equivalent; MRI: magnetic resonance imaging; PA, physical activity; | | | | | | | | |
| ^a^ Continuous variables are expressed as medians (interquartile range), means [standard deviation], and categorical variables are expressed as percentages. | | | | | | | | |

| **Table S2. Association between different types of screentime and risk of dementia, Parkinson’s disease, depression and multimorbidity status in the UK Biobank Study (stratified by age and sex) ^a^** | | | | | | |
| --- | --- | --- | --- | --- | --- | --- |
|  | **Computer use outside of work, hours/day (0 is reference)** | | | **TV viewing time, hours/day (0 to 1 is reference)** | | |
|  | 0.5 to 1 | 2 to 3 | ≥4 | 2 | 3 | ≥4 |
| **Dementia** |  |  |  |  |  |  |
| **Sex** |  |  |  |  |  |  |
| male (n = 214,382) | 0.69 (0.63, 0.75) ^b^ | 0.72 (0.65, 0.80) | 0.76 (0.65, 0.9) | 0.98 (0.87, 1.12) | 1.15 (1.01, 1.30) | 1.31 (1.16, 1.47) |
| female (n = 258,760) | 0.65 (0.59, 0.71) | 0.78 (0.68, 0.88) | 0.76 (0.59, 0.98) | 1.07 (0.93, 1.24) | 1.07 (0.93, 1.23) | 1.22 (1.07, 1.39) |
| **Age** |  |  |  |  |  |  |
| <60 (n = 267,419) | 0.71 (0.60, 0.83) | 0.79 (0.64, 0.97) | 0.86 (0.64, 1.15) | 0.96 (0.77, 1.20) | 1.19 (0.96, 1.48) | 1.51 (1.22, 1.86) |
| ≥60 (n = 205,723） | 0.67 (0.63, 0.72) | 0.73 (0.67, 0.80) | 0.74 (0.63, 0.86) | 1.03 (0.93, 1.15) | 1.08 (0.98, 1.20) | 1.21 (1.10, 1.34) |
|  |  |  |  |  |  |  |
| **Parkinson’s disease** |  |  |  |  |  |  |
| **Sex** |  |  |  |  |  |  |
| male (n = 214,382) | 0.80 (0.72, 0.90) | 0.79 (0.69, 0.91) | 1.01 (0.83, 1.23) | 1.04 (0.89, 1.22) | 1.12 (0.96, 1.31) | 1.26 (1.08, 1.46) |
| female (n = 258,760) | 0.90 (0.78, 1.03) | 1.17 (0.97, 1.41) | 1.38 (1.00, 1.91) | 1.01 (0.83, 1.23) | 0.91 (0.75, 1.11) | 1.02 (0.85, 1.24) |
| **Age** |  |  |  |  |  |  |
| <60 (n = 267,419) | 0.75 (0.61, 0.93) | 0.82 (0.63, 1.06) | 1.33 (0.96, 1.83) | 0.90 (0.71, 1.14) | 0.92 (0.72, 1.19) | 1.08 (0.84, 1.38) |
| ≥60 (n = 205,723） | 0.87 (0.78, 0.96) | 0.91 (0.81, 1.03) | 1.03 (0.85, 1.26) | 1.08 (0.94, 1.24) | 1.08 (0.94, 1.24) | 1.20 (1.05, 1.37) |
|  |  |  |  |  |  |  |
| **Depression** |  |  |  |  |  |  |
| **Sex** |  |  |  |  |  |  |
| male (n = 214,382) | 0.79 (0.74, 0.83) | 0.93 (0.88, 1.00) | 0.99 (0.91, 1.09) | 1.04 (0.97, 1.12) | 1.10 (1.02, 1.19) | 1.33 (1.24, 1.43) |
| female (n = 258,760) | 0.89 (0.85, 0.92) | 1.03 (0.98, 1.08) | 1.11 (1.02, 1.20) | 1.09 (1.04, 1.15) | 1.14 (1.08, 1.20) | 1.34 (1.27, 1.41) |
| **Age** |  |  |  |  |  |  |
| <60 (n = 267,419) | 0.86 (0.82, 0.90) | 1.06 (1.00, 1.12) | 1.07 (0.99, 1.15) | 1.05 (0.99, 1.10) | 1.11 (1.05, 1.17) | 1.41 (1.33, 1.48) |
| ≥60 (n = 205,723） | 0.84 (0.80, 0.88) | 0.90 (0.85, 0.96) | 1.05 (0.95, 1.16) | 1.10 (1.02, 1.18) | 1.11 (1.03, 1.19) | 1.24 (1.16, 1.33) |
|  |  |  |  |  |  |  |
| **Dementia and Depression Multimorbidity** |  |  |  |  |  |  |
| **Sex** |  |  |  |  |  |  |
| male (n = 214,382) | 0.69 (0.55, 0.87) | 0.86 (0.67, 1.10) | 1.02 (0.71, 1.46) | 1.17 (0.84, 1.64) | 1.34 (0.97, 1.86) | 1.73 (1.28, 2.35) |
| female (n = 258,760) | 0.60 (0.49, 0.73) | 0.79 (0.61, 1.03) | 0.48 (0.26, 0.91) | 1.11 (0.82, 1.52) | 1.09 (0.80, 1.49) | 1.29 (0.97, 1.72) |
| **Age** |  |  |  |  |  |  |
| <60 (n = 267,419) | 0.61 (0.44, 0.84) | 0.82 (0.56, 1.19) | 0.73 (0.41, 1.30) | 0.91 (0.59, 1.41) | 1.02 (0.66, 1.58) | 1.45 (0.97, 2.17) |
| ≥60 (n = 205,723） | 0.65 (0.55, 0.77) | 0.82 (0.67, 1.00) | 0.84 (0.59, 1.20) | 1.22 (0.94, 1.60) | 1.25 (0.96, 1.63) | 1.49 (1.17, 1.91) |
|  |  |  |  |  |  |  |
| **Parkinson’s disease and Depression Multimorbidity** |  |  |  |  |  |  |
| **Sex** |  |  |  |  |  |  |
| male (n = 214,382) | 0.49 (0.35, 0.67) | 0.53 (0.36, 0.76) | 0.92 (0.57, 1.48) | 1.02 (0.65, 1.59) | 1.33 (0.87, 2.05) | 1.48 (0.98, 2.23) |
| female (n = 258,760) | 0.73 (0.53, 1.01) | 1.13 (0.75, 1.71) | 0.79 (0.32, 1.95) | 1.02 (0.60, 1.71) | 1.32 (0.80, 2.17) | 1.43 (0.89, 2.31) |
| **Age** |  |  |  |  |  |  |
| <60 (n = 267,419) | 0.61 (0.37, 1.02) | 0.65 (0.34, 1.25) | 0.81 (0.33, 1.97) | 1.40 (0.68, 2.86) | 1.52 (0.73, 3.18) | 2.47 (1.23, 4.96) |
| ≥60 (n = 205,723） | 0.58 (0.45, 0.75) | 0.74 (0.54, 1.01) | 1.04 (0.65, 1.65) | 0.91 (0.62, 1.34) | 1.23 (0.86, 1.77) | 1.25 (0.88, 1.76) |
| ^a^ Obtained by using multivariable Cox regression model. Adjusted for age, sex, body mass index, race, smoking status, alcohol drinking status, education level, visiting friends, living alone, physical activity, Townson depressive index, healthy dietary score, family history of disease (including dementia, PD, and depression), hypertension, diabetes, and computer using time in TV analysis or TV viewing time in computer analysis. | | | | | | |
| ^b^ Hazard ratios (95% confidence interval) (all such values). | | | | | | |

| **Table S3.** **Association between different types of screentime and risk of dementia, Parkinson’s disease, depression and multimorbidity status in the UK Biobank Study (excluded dementia, Parkinson’s disease or depression that occurred in the first 3 years) ^a^** | | | | | | | | |
| --- | --- | --- | --- | --- | --- | --- | --- | --- |
|  | **Computer use outside of work (hours/day)** | | | | **TV viewing time (hours/day)** | | | |
|  | 0 | 0.5 to 1 | 2 to 3 | ≥4 | 0 to 1 | 2 | 3 | ≥4 |
| **Dementia** |  |  |  |  |  |  |  |  |
| No. of dementia | 2,571 | 1,981 | 927 | 241 | 732 | 1,151 | 1,347 | 2,490 |
| Person years | 1,535,586 | 2,859,738 | 951,689 | 285,231 | 1,172,848 | 1,527,664 | 1,334,822 | 1,596,910 |
| Incidence per 1000 person years | 1.67 | 0.69 | 0.97 | 0.84 | 0.62 | 0.75 | 1.01 | 1.56 |
| Model 1 | 1.00 (reference) | 0.57 (0.54, 0.61) ^b^ | 0.66 (0.61, 0.71) | 0.71 (0.62, 0.81) | 1.00 (reference) | 1.06 (0.97, 1.17) | 1.18 (1.08, 1.29) | 1.50 (1.38, 1.63) |
| Model 2 | 1.00 (reference) | 0.69 (0.65, 0.73) | 0.76 (0.70, 0.82) | 0.78 (0.68, 0.89) | 1.00 (reference) | 1.05 (0.95, 1.15) | 1.12 (1.02, 1.22) | 1.27 (1.17, 1.39) |
|  |  |  |  |  |  |  |  |  |
| **Parkinson’s disease** |  |  |  |  |  |  |  |  |
| No. of Parkinson’s disease | 902 | 1,109 | 478 | 144 | 416 | 595 | 641 | 981 |
| Person years | 1,538,184 | 2,859,828 | 951,633 | 285,115 | 1,172,867 | 1,527,687 | 1,335,483 | 1,598,723 |
| Incidence per 1000 person years | 0.59 | 0.39 | 0.50 | 0.51 | 0.35 | 0.39 | 0.48 | 0.61 |
| Model 1 | 1.00 (reference) | 0.85 (0.78, 0.93) | 0.88 (0.79, 0.98) | 1.06 (0.89, 1.26) | 1.00 (reference) | 0.99 (0.87, 1.12) | 1.03 (0.91, 1.17) | 1.12 (0.99, 1.26) |
| Model 2 | 1.00 (reference) | 0.86 (0.79, 0.95) | 0.87 (0.78, 0.98) | 1.02 (0.85, 1.22) | 1.00 (reference) | 1.01 (0.89, 1.15) | 1.08 (0.95, 1.22) | 1.15 (1.02, 1.30) |
|  |  |  |  |  |  |  |  |  |
| **Depression** |  |  |  |  |  |  |  |  |
| No. of depression | 6,543 | 8,191 | 3,353 | 1,075 | 3,030 | 4,504 | 4,442 | 7,186 |
| Person years | 1,500,251 | 2,814,139 | 932,468 | 278,286 | 1,155,650 | 1,502,255 | 1,310,452 | 1,556,787 |
| Incidence per 1000 person years | 4.36 | 2.91 | 3.60 | 3.86 | 2.62 | 3.00 | 3.39 | 4.62 |
| Model 1 | 1.00 (reference) | 0.70 (0.68, 0.73) | 0.88 (0.84, 0.92) | 0.95 (0.89, 1.02) | 1.00 (reference) | 1.10 (1.05, 1.15) | 1.21 (1.16, 1.27) | 1.59 (1.53, 1.67) |
| Model 2 | 1.00 (reference) | 0.86 (0.83, 0.89) | 1.00 (0.96, 1.05) | 1.04 (0.97, 1.11) | 1.00 (reference) | 1.08 (1.03, 1.14) | 1.13 (1.08, 1.19) | 1.33 (1.27, 1.39) |
|  |  |  |  |  |  |  |  |  |
| **Dementia and Depression Multimorbidity** |  |  |  |  |  |  |  |  |
| No. of disease | 458 | 317 | 180 | 44 | 116 | 191 | 227 | 465 |
| Person years | 1,540,808 | 2,863,547 | 953,574 | 285,734 | 1,174,354 | 1,529,896 | 1,337,463 | 1,601,950 |
| Incidence per 1000 person years | 0.30 | 0.11 | 0.19 | 0.15 | 0.10 | 0.12 | 0.17 | 0.29 |
| Model 1 | 1.00 (reference) | 0.51 (0.44, 0.58) | 0.70 (0.58, 0.83) | 0.78 (0.58, 1.05) | 1.00 (reference) | 1.19 (0.94, 1.50) | 1.31 (1.04, 1.64) | 1.88 (1.53, 2.32) |
| Model 2 | 1.00 (reference) | 0.65 (0.56, 0.76) | 0.84 (0.7, 1.01) | 0.87 (0.65, 1.18) | 1.00 (reference) | 1.18 (0.94, 1.50) | 1.22 (0.97, 1.54) | 1.52 (1.22, 1.89) |
|  |  |  |  |  |  |  |  |  |
| **Parkinson’s disease and Depression Multimorbidity** |  |  |  |  |  |  |  |  |
| No. of disease | 158 | 130 | 63 | 19 | 50 | 71 | 99 | 150 |
| Person years | 1,541,378 | 2,863,874 | 953,717 | 285,748 | 1,174,532 | 1,530,065 | 1,337,663 | 1,602,457 |
| Incidence per 1000 person years | 0.10 | 0.05 | 0.07 | 0.07 | 0.04 | 0.05 | 0.07 | 0.09 |
| Model 1 | 1.00 (reference) | 0.56 (0.44, 0.70) | 0.68 (0.51, 0.90) | 0.95 (0.62, 1.46) | 1.00 (reference) | 1.01 (0.72, 1.44) | 1.28 (0.91, 1.78) | 1.49 (1.09, 2.04) |
| Model 2 | 1.00 (reference) | 0.62 (0.49, 0.79) | 0.72 (0.54, 0.97) | 0.97 (0.63, 1.50) | 1.00 (reference) | 1.06 (0.75, 1.50) | 1.33 (0.95, 1.87) | 1.45 (1.04, 2.01) |
| ^a^ Obtained by using multivariable Cox regression model. | | | | | | | | |
| ^b^ Hazard ratios (95% confidence interval) (all such values). | | | | | | | | |
| Model 1 was adjusted for age, sex, and body mass index. | | | | | | | | |
| Model 2 was additionally adjusted for race, smoking status, alcohol drinking status, education level, visiting friends, living alone, physical activity, Townson depressive index, healthy dietary score, family history of disease (including dementia, PD, and depression), hypertension, diabetes, and computer using time in TV analysis or TV viewing time in computer analysis. | | | | | | | | |

**S Figures 1** **The associations between different types of screen time and incident dementia, Parkinson’s disease, depression and** **multimorbidity status using a restricted cubic spline regression model.** (A1) computer using and dementia. (A2) TV viewing and dementia. (B1) computer using and PD. (B2) TV viewing and PD. (C1) computer using and depression. (C2) TV viewing and depression. (D1) computer using and dementia and depression multimorbidity. (D2) TV viewing and dementia and depression multimorbidity. (E1) computer using and PD and depression multimorbidity. (E2) TV viewing and PD and depression multimorbidity. The 95% CIs of the adjusted HRs are represented by the shaded area. Restricted cubic spline model is adjusted for age, sex, BMI, race, smoking status, alcohol drinking status, education level, visiting friends, living alone, physical activity, Townson depressive index, healthy dietary score, family history of disease (including dementia, PD, and depression), hypertension, diabetes, and computer using time in TV analysis or TV viewing time in computer analysis. BMI, body mass index; HR, hazard ratio; PD, Parkinson’s disease.

**A1**

P non-linear <0.0001

**
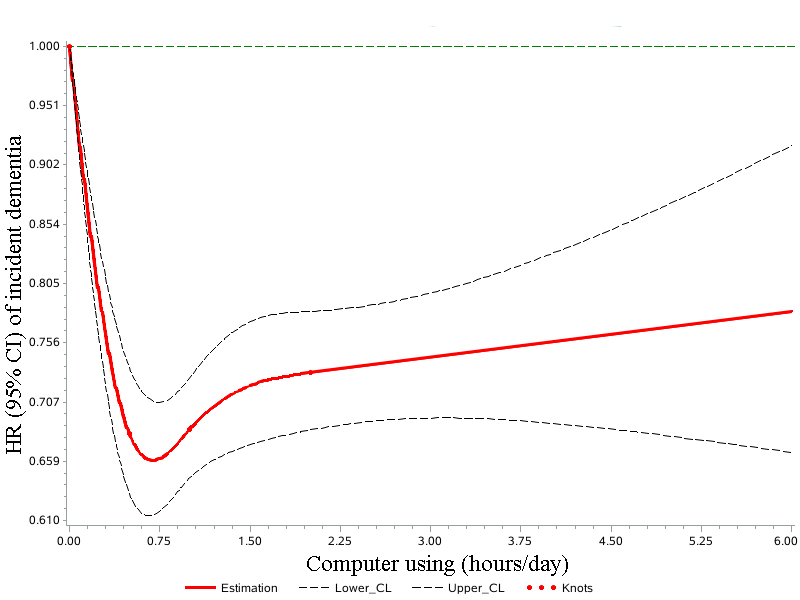
**

**A2**

P non-linear <0.0001

**
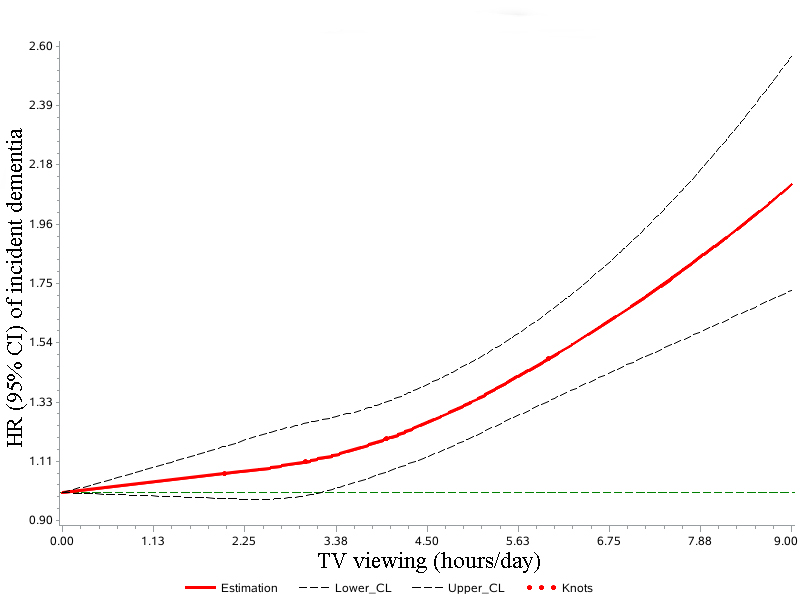
**

**B1**

P non-linear =0.0004

**
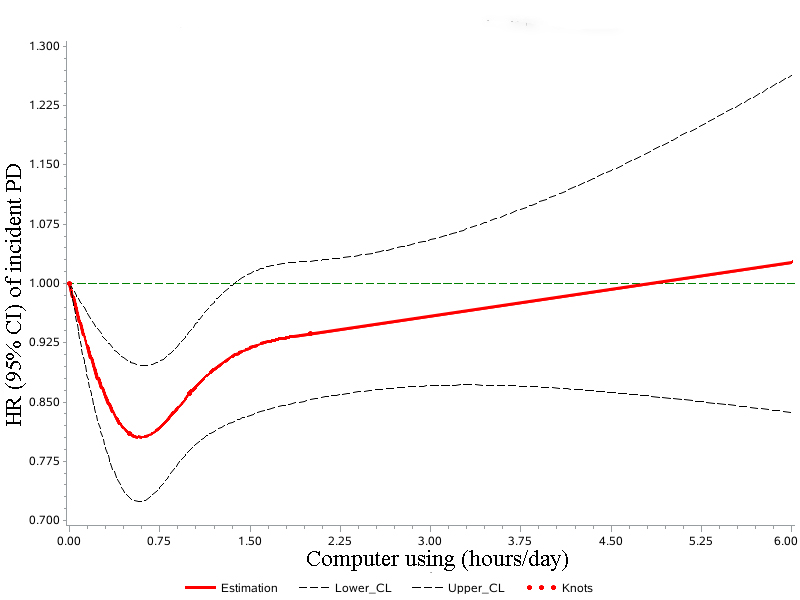
**

**B2**

P non-linear =0.0003

**
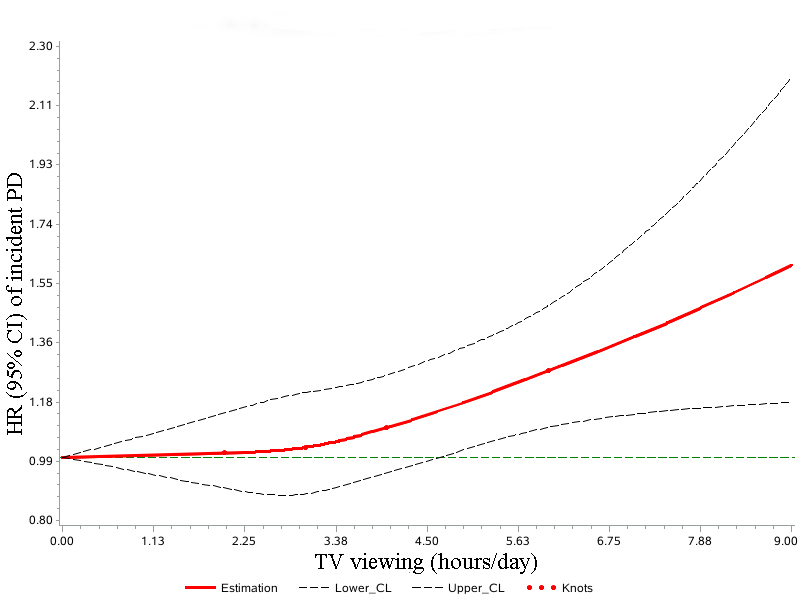
**

**C1**

P non-linear <0.0001

**
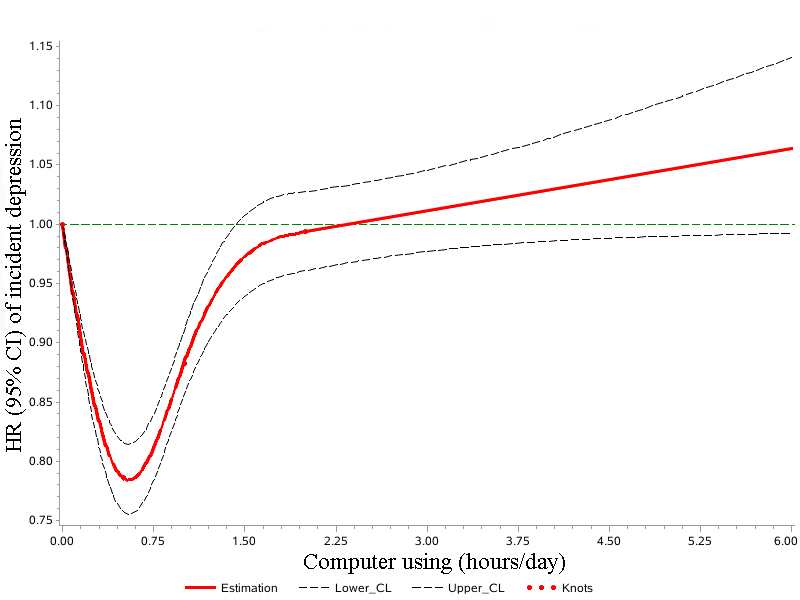
**

**C2**

P non-linear <0.0001

**
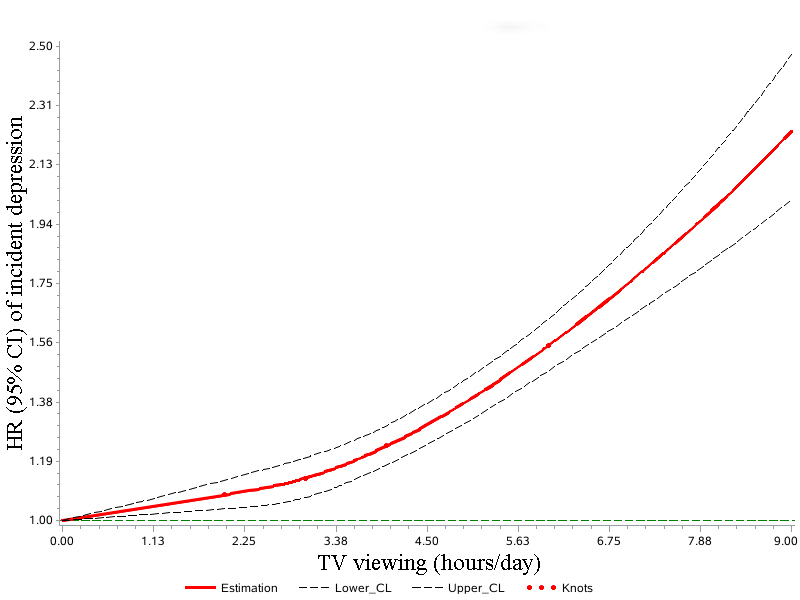
**

**D1**

P non-linear <0.0001

**
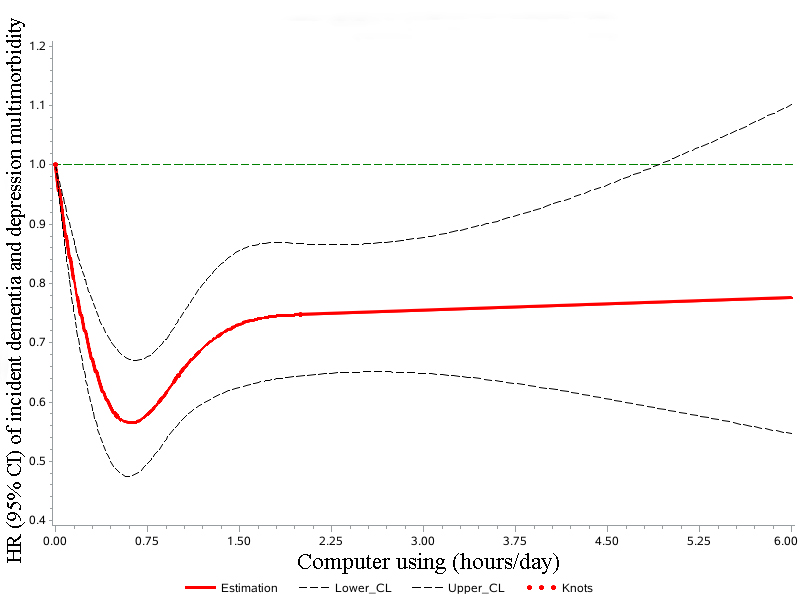
**

**D2**

P non-linear <0.0001

**
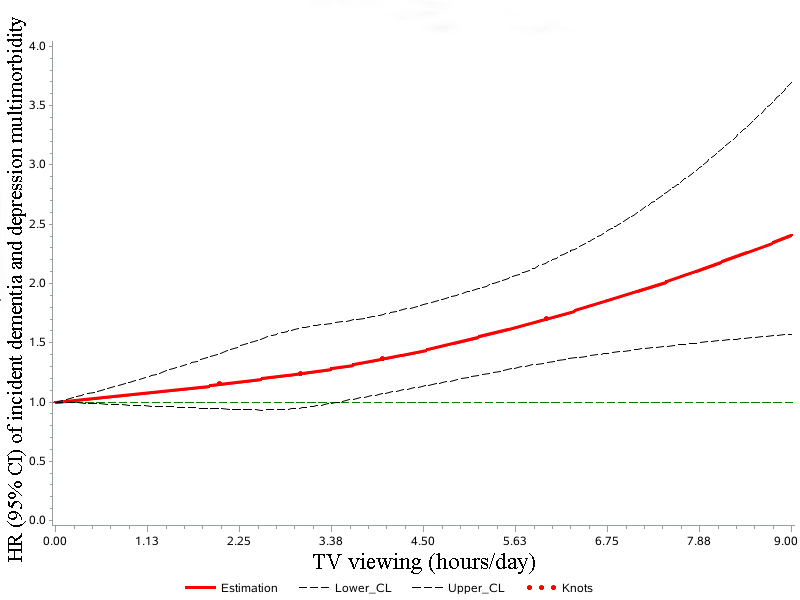
**

**E1**

P non-linear <0.0001

**
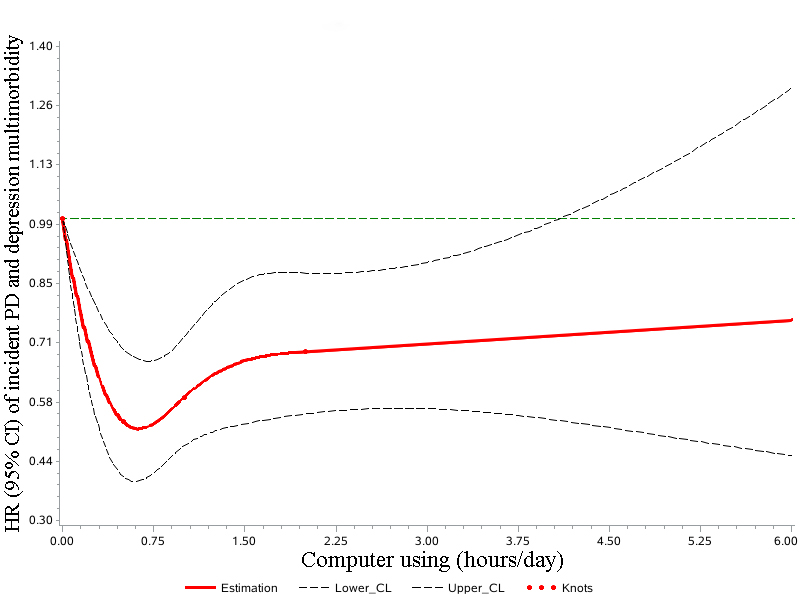
**

**E2**

P non-linear <0.0001

**
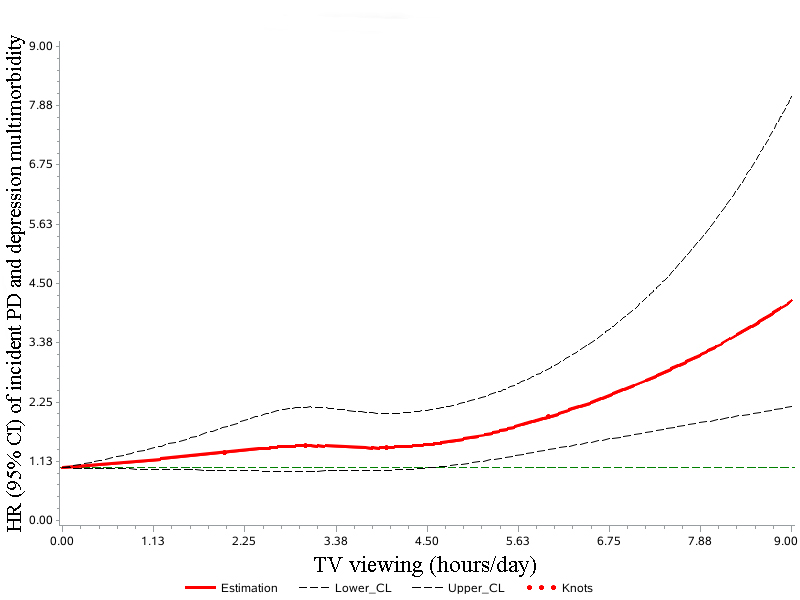
**

**S Figures 2 The associations between different types of screen time with brain MRI indices using a restricted cubic spline regression model.** (A1) computer using and WMH. (A2) computer using and hippocampal volume. (A3) computer using and total brain volume. (A4) computer using and total white matter volume. (A5) computer using and total gray matter volume. (B1) TV viewing and WMH. (B2) TV viewing and hippocampal volume. (B3) TV viewing and total brain volume. (B4) TV viewing and total white matter volume. (B5) TV viewing and PD and total gray matter volume. The 95% CIs of the adjusted beta are represented by the shaded area. Restricted cubic spline model is adjusted for age, sex, BMI, race, smoking status, alcohol drinking status, education level, visiting friends, living alone, physical activity, Townson depressive index, healthy dietary score, family history of disease (including dementia, PD, and depression), hypertension, diabetes, and computer using time in TV analysis or TV viewing time in computer analysis. BMI, body mass index; PD, Parkinson’s disease.

**A1**

P non-linear =0.02

**
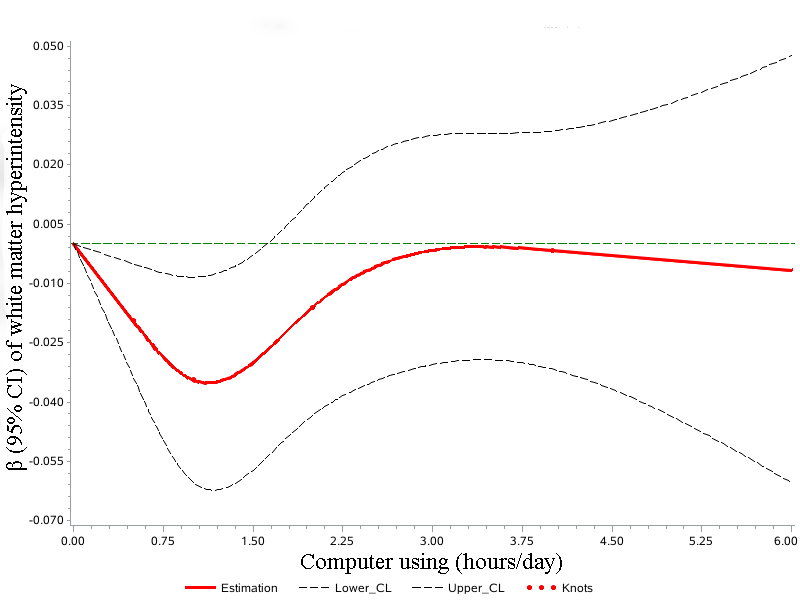
**

**A2**

P non-linear <0.001


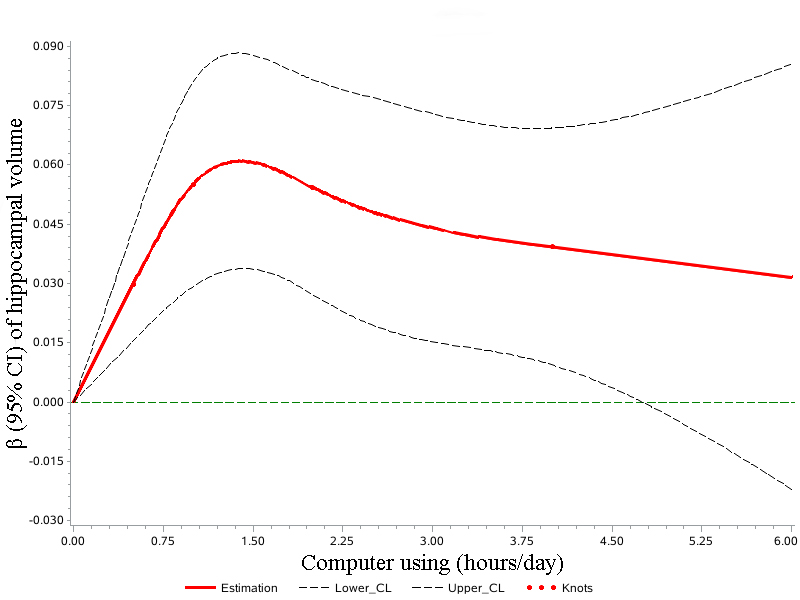


**A3**

P non-linear =0.37


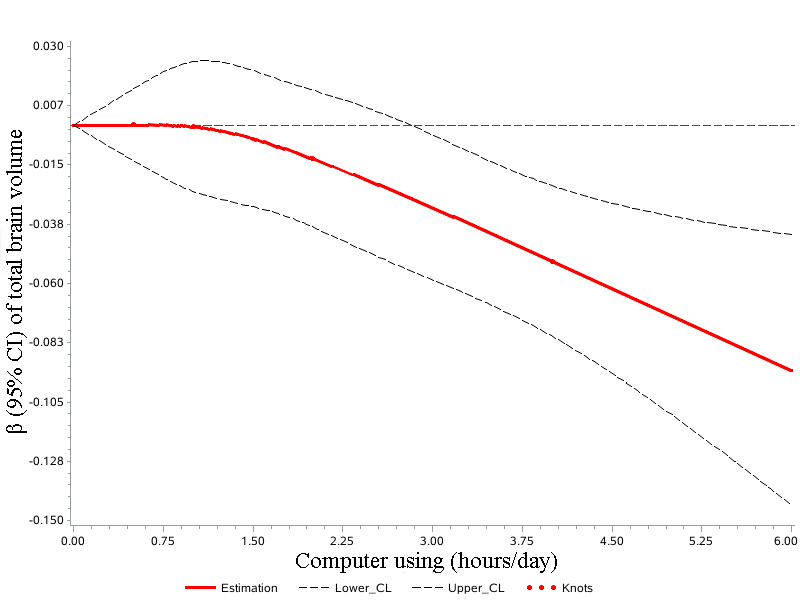


**A4**

P non-linear =0.68


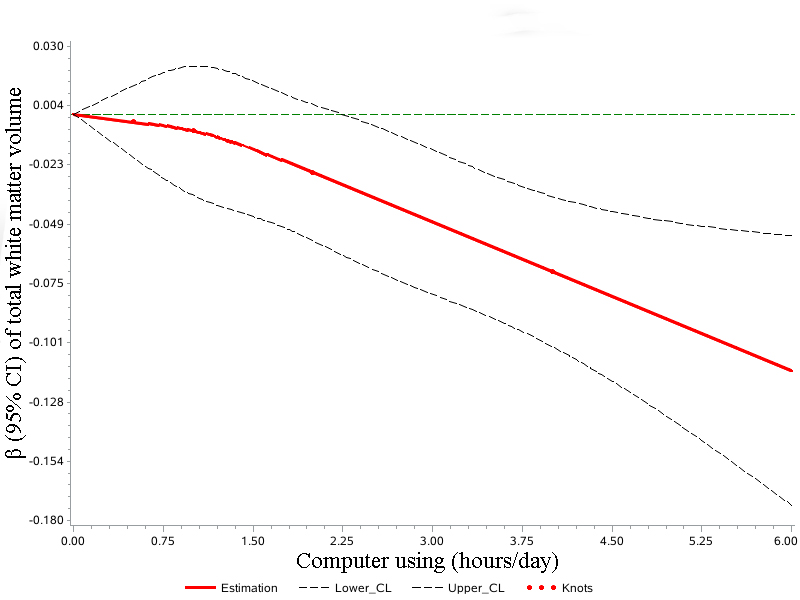


**A5**

P non-linear =0.35

**
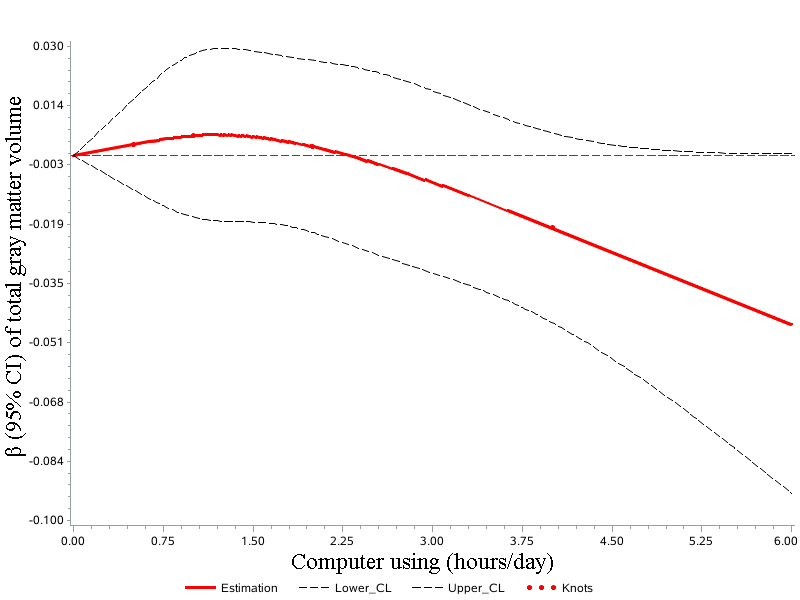
**

**B1**

P non-linear =0.42

**
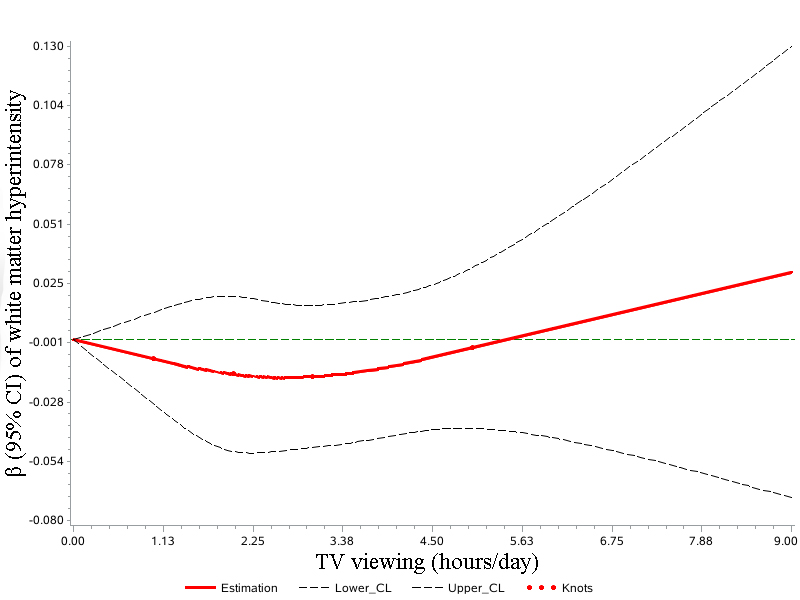
**

**B2**

P non-linear <0.01

**
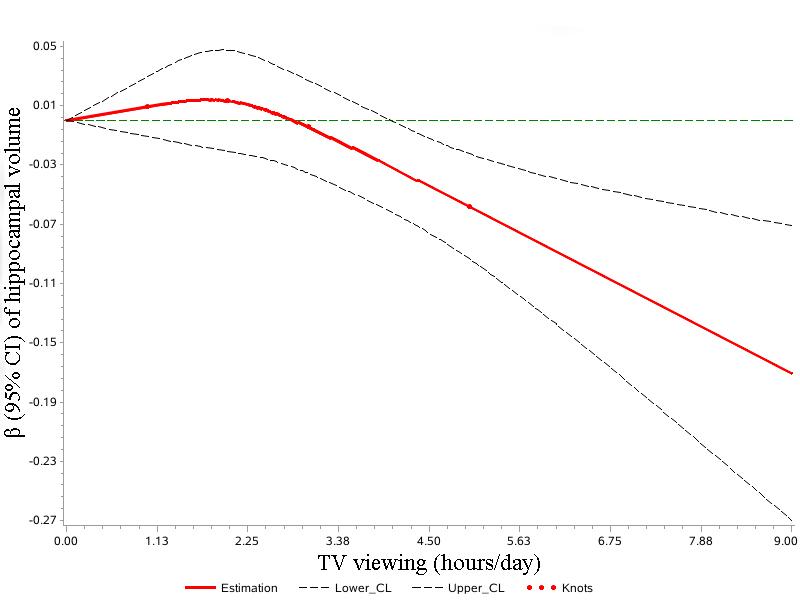
**

**B3**

P non-linear <0.001

**
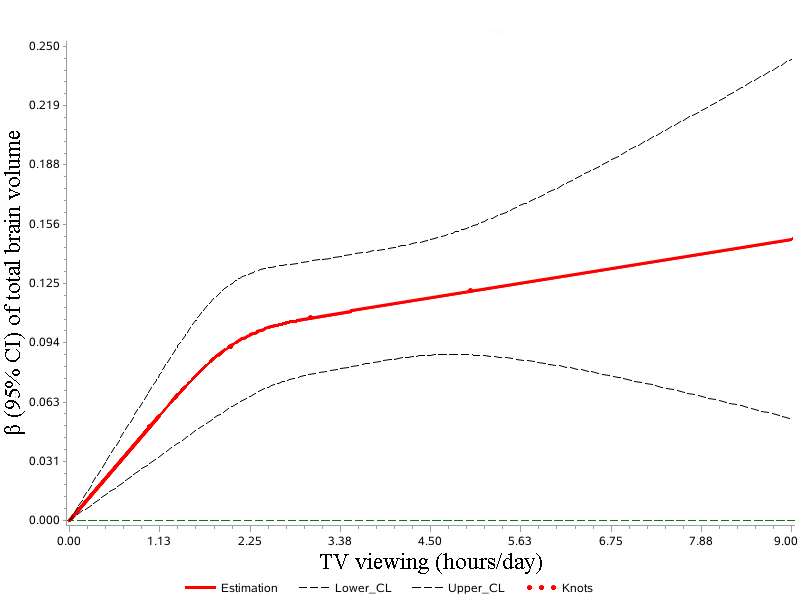
**

**B4**

P non-linear =0.42

**
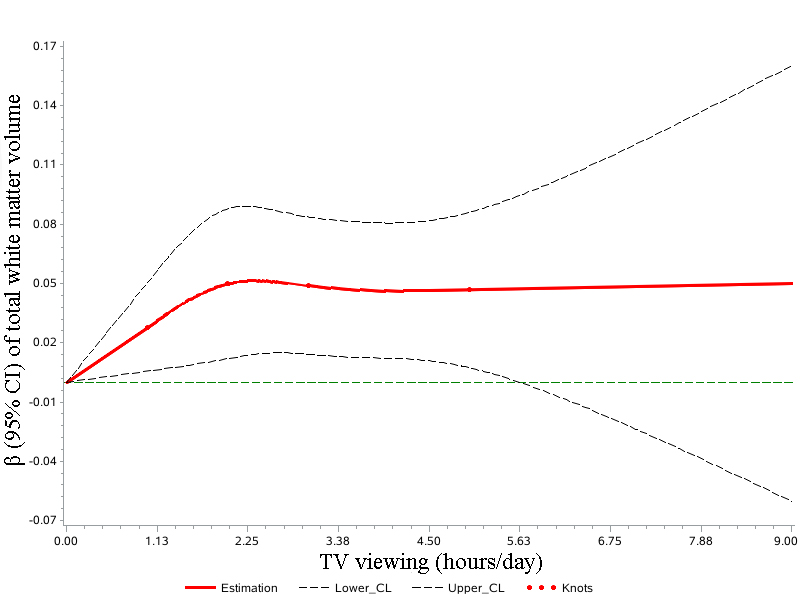
**

**B5**

P non-linear <0.001

**
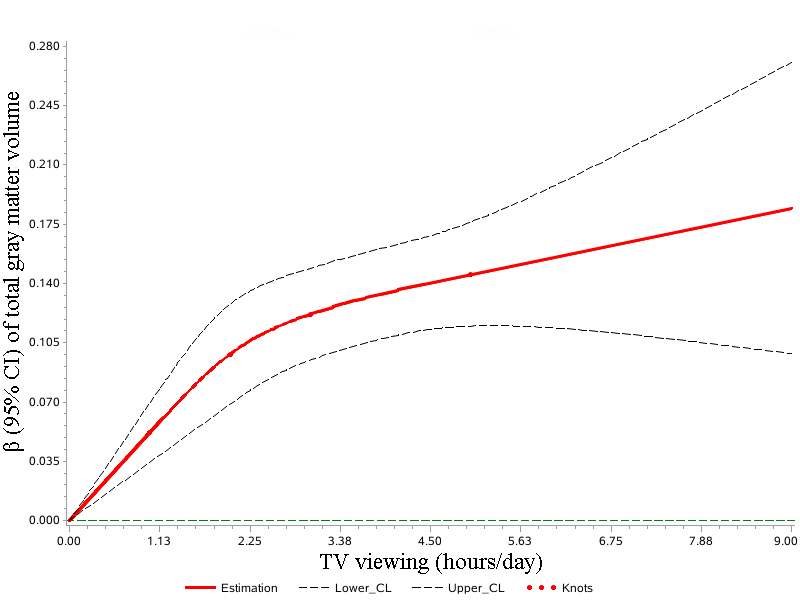
**

**Reference**

1. Sudlow C, Gallacher J, Allen N, Beral V, Burton P, Danesh J, Downey P, Elliott P, Green J, Landray M *et al*: **UK biobank: an open access resource for identifying the causes of a wide range of complex diseases of middle and old age**. *PLoS Med* 2015, **12**(3):e1001779.

2. **Guidelines for data processing and analysis of IPAQ** [<https://biobank.ndph.ox.ac.uk/showcase/ukb/docs/ipaq_analysis.pdf>]

3. Townsend P, Phillimore P, Beattie A: **Health and Deprivation: Inequality and the North. Croom Helm, Bristol**. 1988.

4. Lee DH, Keum N, Hu FB, Orav EJ, Rimm EB, Willett WC, Giovannucci EL: **Predicted lean body mass, fat mass, and all cause and cause specific mortality in men: prospective US cohort study**. *BMJ* 2018, **362**:k2575.

5. Wu Y, Zhang D, Kang S: **Physical activity and risk of breast cancer: a meta-analysis of prospective studies**. *Breast Cancer Res Treat* 2013, **137**(3):869-882.

6. Mekary RA, Lucas M, Pan A, Okereke OI, Willett WC, Hu FB, Ding EL: **Isotemporal substitution analysis for physical activity, television watching, and risk of depression**. *Am J Epidemiol* 2013, **178**(3):474-483.
